# Interpreting regulatory mechanisms of Hippo signaling through a deep learning sequence model

**DOI:** 10.1016/j.xgen.2025.100821

**Published:** 2025-04-01

**Authors:** Khyati Dalal, Charles McAnany, Melanie Weilert, Mary Cathleen McKinney, Sabrina Krueger, Julia Zeitlinger

**Affiliations:** 1 Stowers Institute for Medical Research, Kansas City, MO, USA; 2 Department of Pathology & Laboratory Medicine, The University of Kansas Medical Center, Kansas City, KS, USA

**Keywords:** Hippo signaling pathway, TEAD4, YAP1, transcription factors, ChIP-nexus, BPNet, interpretable deep learning, enhancer redesign, mouse trophoblast stem cells, molecular dynamics, CRISPR-Cas9

## Abstract

Signaling pathway components are well studied, but how they mediate cell-type-specific transcription responses is an unresolved problem. Using the Hippo pathway in mouse trophoblast stem cells as a model, we show that the DNA binding of signaling effectors is driven by cell-type-specific sequence rules that can be learned genome wide by deep learning models. Through model interpretation and experimental validation, we show that motifs for the cell-type-specific transcription factor TFAP2C enhance TEAD4/YAP1 binding in a nucleosome-range and distance-dependent manner, driving synergistic enhancer activation. We also discovered that *Tead double* motifs are widespread, highly active canonical response elements. Molecular dynamics simulations suggest that TEAD4 binds them cooperatively through surprisingly labile protein-protein interactions that depend on the DNA template. These results show that the response to signaling pathways is encoded in the *cis*-regulatory sequences and that interpreting the rules reveals insights into the mechanisms by which signaling effectors influence cell-type-specific enhancer activity.

## Highlights

• Interpretable deep learning uncovers *cis*-regulatory rules downstream of Hippo signaling
• YAP1 binding serves as a proxy for predicting cell-type-specific enhancer activity
• TFAP2C enhances TEAD4/YAP1 binding within nucleosome range, dependent on distance
• Two TEAD4s cooperate on the *Tead double* motif through transient, labile interactions

## Graphical abstract

**Figure:**
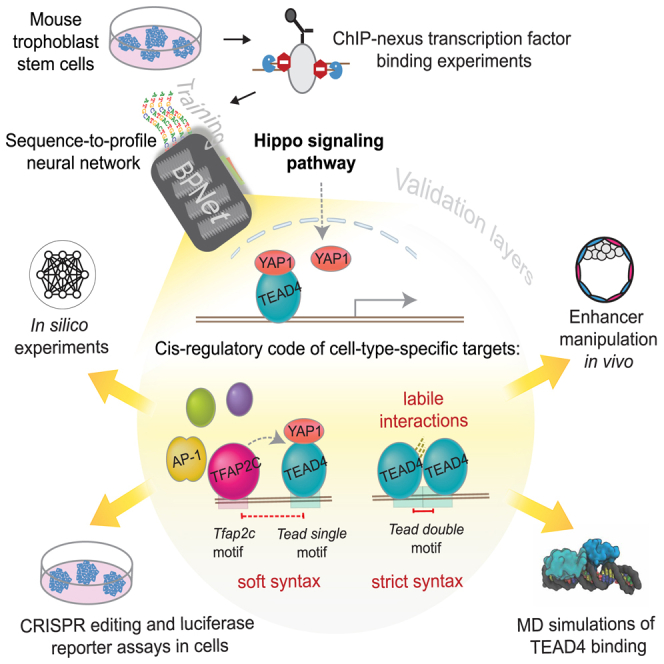

## Teaser

Dalal et al. show how AI can be used to systematically uncover the sequence rules that guide how signaling pathways interact with DNA inside cells. This work provides a new lens for discovering the cooperative mechanisms that shape cell-type-specific gene expression in response to cellular signaling.

## Introduction

Signaling pathways are critical for cell fate decisions during development, the generation of cell types *in vitro,* and therapeutic interventions, which often target-specific signaling pathways.^1^ The signaling components and transcription factors (TFs) that function as canonical effectors of a pathway are typically well studied. Such signaling effectors recognize specific DNA sequence motifs either directly or find obligate partner TFs with DNA binding specificity. However, signaling pathways are reiteratively used during development, and which *cis*-regulatory sequences are bound by these TFs and become active enhancers that regulate target genes is highly complex and poorly understood.^2^^,^^3^ Thus, signaling pathways are critical for gene regulation, but their target specificity is one of the least understood areas of enhancer biology, making it difficult to predict the activity of enhancers or modify their function during development through targeted mutations.^3^^,^^4^^,^^5^^,^^6^^,^^7^

Here, we hypothesized that the binding of signaling effectors is encoded in the *cis*-regulatory sequences. This hypothesis is supported by studies on individual enhancers showing that the target gene specificity of a signaling pathway depends on cell-type-specific TFs.^2^^,^^8^ Furthermore, genetic experiments showed that cell-type-specific TFs help determine where signaling effectors bind.^9^^,^^10^^,^^11^^,^^12^^,^^13^ However, there is no systematic approach to capture the genome-wide *cis*-regulatory sequence rules, identify relevant cell-type-specific TFs, and characterize the molecular mechanisms by which they influence enhancer activation.^14^

Capturing genome-wide *cis*-regulatory sequence rules in a cell-type-specific manner is an ideal task for a deep learning model. By learning to predict TF binding data from DNA sequence, convolutional neural networks learn complex sequence rules in an unbiased way, and the rules can be extracted from the trained models using interpretation tools.^15^^,^^16^^,^^17^^,^^18^^,^^19^^,^^20^^,^^21^ A key advantage is the inherent predictive accuracy and the genome-wide applicability of the learned sequence rules, something that cannot be achieved by studying motifs on individual enhancers experimentally or by analyzing motif compositions using traditional methods.

Predictive accuracy is the first step during training, where the model learns to predict TF binding profiles from sequence. The genome-wide applicability is ensured by testing the performance on withheld data that the model has not seen during training. Another advantage is that the sequence rules are learned *de novo* in an inherently combinatorial way on large amounts of data. Only upon achieving accurate predictions can the model be interpreted to extract the learned rules. Such models not only learn TF motifs, but also measurements of their relative affinities and the syntax rules, and thus the distance relationships by which motifs cooperate with each other.^15^^,^^17^^,^^20^^,^^22^^,^^23^

We reasoned that interpretable deep learning might identify which cell-type-specific TF motifs contribute to the response to signaling, and that the motif syntax rules could pinpoint to potential molecular mechanisms. TF cooperativity is often studied with the assumption that TFs interact with each other through protein-protein interactions, exemplified by the pioneering work of the interferon-beta enhanceosome.^24^^,^^25^^,^^26^ For such protein-protein interactions to occur, two motifs have to be well positioned at close distances, which should be reflected in the syntax rules. On the other hand, such strictly spaced motifs are not frequently observed in the genome, raising the question of whether TF binding cooperativity often occurs through more flexible motif syntax.^27^^,^^28^^,^^29^ Indeed, deep learning models suggest the existence of a soft motif syntax, which occurs at variable motif distances within ∼150 bp, with stronger TF cooperativity predicted at closer distances.^15^^,^^20^

Whether such syntax rules exist for signaling effectors has previously been difficult to decipher. ChIP-seq binding data tend to be of low resolution and display low levels of signal when the TF binds indirectly through a partner TF.^10^^,^^11^^,^^13^ Likewise, individually manipulating enhancer sequences *in vivo* limits throughput, and the effects can be difficult to interpret since they may be enhancer-specific or caused by the inadvertent disruption of other important sequences.^30^^,^^31^ Large-scale reporter assays, on the other hand, have produced conflicting results on whether motif syntax is important and, if so, their effects have only been measured for short motif distances and not been mechanistically analyzed.^16^^,^^27^^,^^32^^,^^33^^,^^34^^,^^35^^,^^36^^,^^37^ For these reasons, TF binding cooperativity downstream of signaling pathways has not been systematically studied from a sequence perspective.

To test whether the binding of signaling effectors is sequence encoded and follows syntax rules, we performed TF binding experiments at the highest resolution and leveraged our previously developed deep learning model BPNet to predict the data at base resolution from genomic sequences of 1 kb.^15^^,^^20^^,^^22^^,^^38^^,^^39^ This approach has high predictive accuracy and optimally resolves sequence rules between closely spaced motifs within enhancers.^15^ Since the model does not predict enhancer activity or target genes, we evaluated and validated these downstream aspects using traditional methods.

As a model system for our approach, we studied the Hippo signaling pathway in mouse trophoblast stem cells (TSCs). Hippo signaling is critical for specifying trophoblast versus inner cell mass cell fate in the early mouse embryo.^40^^,^^41^^,^^42^^,^^43^^,^^44^^,^^45^ When cells of the embryo sense that they are facing the outside, i.e., less cell density, they polarize and inactivate the Hippo pathway. This causes YAP1/TAZ to translocate to the nucleus and bind to TEAD4, which, like all TEAD family members, binds to a consensus *Tead* motif.^41^^,^^43^^,^^45^^,^^46^ Other TFs known to be important for TSC identity include CDX2, TFAP2C, and GATA3.^47^^,^^48^^,^^49^^,^^50^^,^^51^^,^^52^^,^^53^^,^^54^ TSCs are therefore an ideal system to dissect the interactions between Hippo signaling effectors and cell-type-specific TFs in enhancer activation.

Here, we show that the binding of the signaling effectors YAP1/TEAD4 is specified by *cis*-regulatory sequence rules that apply genome-wide. We identified thousands of novel active enhancers in TSCs and show that their activity is driven by YAP1, which binds DNA with the help of cell-type-specific TFs such as TFAP2C. While the *Tfap2c-Tead* motif synergy follows a soft motif syntax, we also identified the *Tead double* motif as mediating strong cooperativity through strict syntax. Based on our molecular dynamics (MD) simulations, the strict distance between the two *Tead* motifs is required for two TEAD4 to engage in labile protein-protein interactions, suggesting that the TEAD4 effector complex is assembled on DNA with the help of Hippo signaling. This demonstrates how deep learning models can uncover precise sequence rules and potential mechanisms by which signaling effectors bind in the genome to produce cell-type-specific effects.

## Results

### A deep learning model reveals combinatorial binding motifs for Hippo TFs

**Figure 1:**
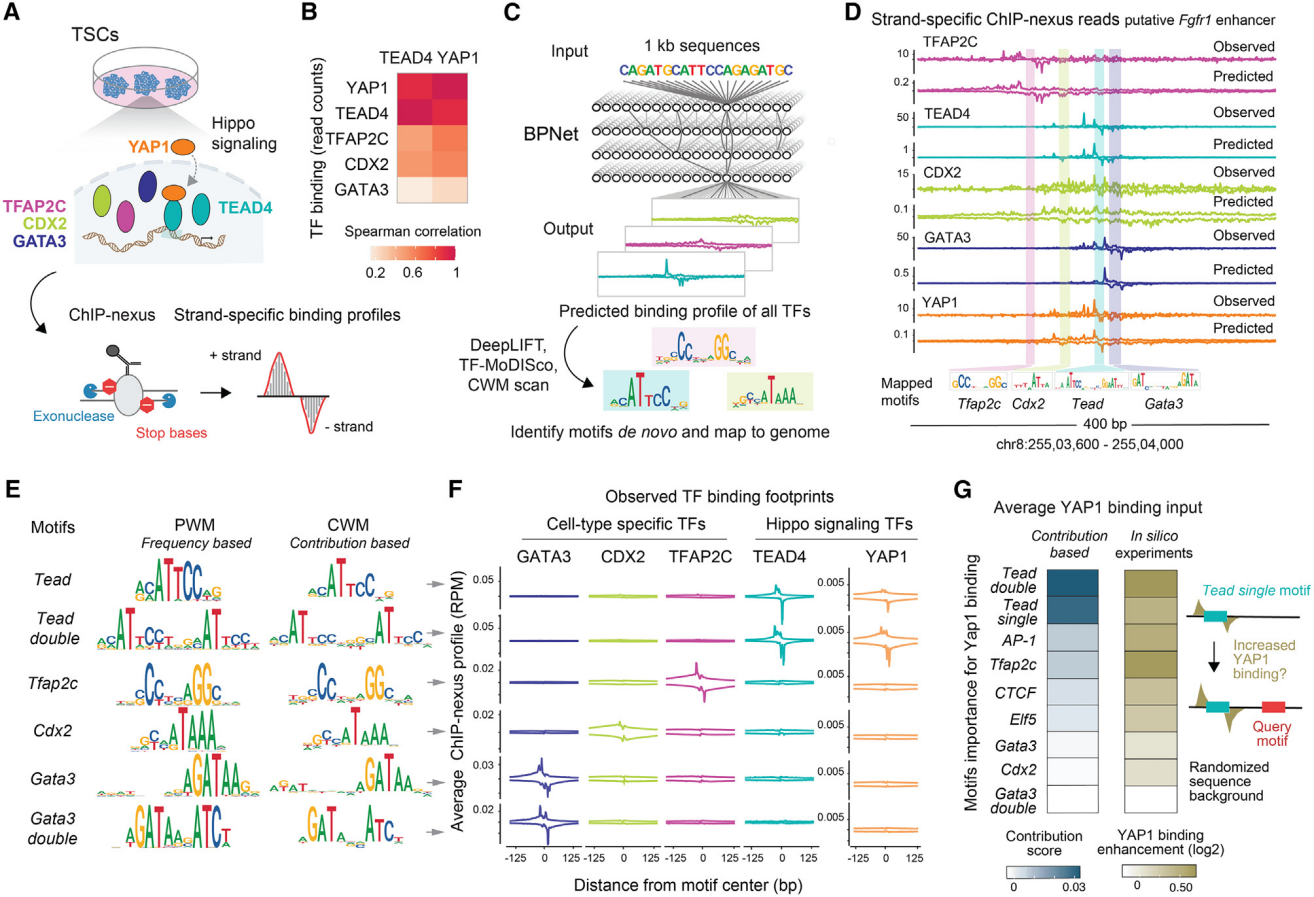
BPNet suggests combinatorial binding motifs for Hippo TFs (A) Experimental design to map high-resolution binding of signaling and cell-type-specific TFs in mouse TSCs. (B) Spearman correlations of the ChIP-nexus read counts between TFs at non-promoter binding regions show that YAP1 and TEAD4 binding are highly correlated. (C) Schematic of the multi-task BPNet model, trained to predict ChIP-nexus TF binding from DNA sequence, and the interpretation tools that identify and map contributing motifs for each TF. (D) At the *Fgfr1* enhancer (mm10-chr8:25503600-2550400) not seen during model training, observed and BPNet-predicted base-resolution binding are visibly similar for each TF (+, strand on top; −, strand below). Quantifications of this similarity by Jensen-Shannon distance (0 = perfect concordance, 1 = no similarity, shown globally in Figure S1D) give values of 0.31 (TFAP2C), 0.32 (TEAD4), 0.32 (CDX2), 0.30 (GATA3), and 0.43 (YAP1). BPNet-mapped motifs are shown below. (E) Learned motifs are shown as frequency-based position weight matrix (PWM) and contribution weight matrix (CWM), where the base height reflects the contribution to the TF binding predictions. (F) Average ChIP-nexus binding footprints of all TFs at BPNet-mapped motifs, shown as reads per million (RPM), with values on + strand on top and − strand below. Sharp footprints typically indicate direct binding of the TF to the motif. YAP1 has sharp footprints on the *Tead* motifs despite binding indirectly. (G) The importance of motifs toward YAP1 binding was assessed by two interpretation methods, one based on the contribution scores from genomic instances (blue on the left) and one based on rules derived *in silico* without genomic context (olive on the right). Contribution scores are derived by DeepLIFT relative to a dinucleotide shuffled control sequence on a log scale, thus have no unit. In the second method (right plot), motifs are injected many times into a randomized background that contains a *Tead single* motif (ACATTCCTG) within 150 bp. The average predicted YAP1 binding enhancement over no added query motif is calculated.

We generated genome-wide, high-resolution binding data for two Hippo signaling effectors (TEAD4 and YAP1) and for potential TSC-specific partner TFs (CDX2, TFAP2C, and GATA3) by using a ChIP-exo technique called ChIP-nexus,^38^ in which an exonuclease step generates narrow and sharp binding footprints (Figures 1A and S1A). We used TSCs derived from mouse blastocysts^55^ and confirmed that they retain features of endogenous trophectoderm (TE) cells by reintegrating them into the TE layer of blastocyst embryos in an aggregation assay (Figure S1B). The ChIP-nexus binding data revealed that YAP1 and TEAD4 were more correlated with each other than any other TF pair (Figure 1B), consistent with YAP1 binding to DNA through TEAD4.^56^^,^^57^^,^^58^

We then trained the deep learning model BPNet to predict the base-resolution binding profiles of all TFs from 187,775 reproducibly bound genomic regions (Figure 1C) by separating chromosomes into training, validation, and test groups to confirm model accuracy. We also performed cross-validation on different chromosome combinations to ensure model stability (Figure S1F). For all TFs, we obtained high prediction accuracy for the read counts, as well as profiles and footprint positions on a par with the similarity between replicate experiments (Figures 1D and S1C–S1E). Examples include the putative enhancers of important trophoblast genes such as *Fgfr1*^43^^,^^59^ (Figure 1D), *Amotl2*, *Pard3b*, and *Krt8/18*^60^^,^^61^^,^^62^ (Figures S1G and S1I). These results show that the model learned general rules for this cell type to predict TF binding anywhere in the genome from sequence alone.

To understand which motifs contribute to the binding of the signaling effectors TEAD4 and YAP1, we extracted *de novo* learned motifs. Using an attribution method,^63^ we assigned contribution scores to all bases in the input sequences and then summarized learned motifs as a contribution weight matrix (CWM).^64^ The CWM motifs were then used to map “contributing” motif instances in each genomic region (Figure 1C). These mapped motifs were highly congruent with experimentally observed and predicted TF footprints (Figures 1D and S1G–S1I).

Among the discovered motifs were the known consensus motifs of the profiled TFs (Figure 1E) and two unexpected motifs: a strictly spaced *Tead double* motif and a strictly spaced *Gata3 double* motif (Figure 1E). These motifs are directly bound by TEAD4 and GATA3, respectively, as confirmed by the sharpness of the ChIP-nexus footprints (Figure 1F). YAP1 also showed sharp binding footprints on the *Tead* motifs, suggesting a tight physical association between YAP1 and TEAD4 on DNA. Given YAP1’s stronger dependency on Hippo signaling, we focused on understanding how YAP1 binding is influenced by other TFs. Notably, the model learned motifs for TFs that we did not profile, including JUN-FOS (AP-1), CTCF, and ELF5 (Figure S1F).

We then used two complementary model interpretation methods^15^ to measure whether and how much, on average, each motif influences YAP1 binding (Figure 1G). The first method uses the contribution scores of mapped motifs from genomic regions (Figure 1G, left) and measures each motif’s influence across all observed instances *in vivo*. The second method uses *in silico* experiments to measure each motif’s influence in a randomized sequence background (Figure 1G, right), and thus tests the learned rules in isolation without the complexity of genomic sequences. We injected a *Tead single* motif with or without another motif and let the model predict how much any given motif enhanced the binding of YAP1 to the *Tead single* motif. Both methods revealed similar results, providing internal validation of our model interpretation.

The *Tead* motifs, which we will refer to as *Tead single* and *Tead double* motifs, were both important as expected, but the *AP-1* and *Tfap2c* motifs also had a sizable contribution to YAP1 binding (Figure 1G). AP-1 is present in many cell types and has previously been shown to cooperate with TEAD and YAP in cancer cell lines,^65^^,^^66^^,^^67^^,^^68^ confirming the model’s predictions. However, we also identified a strong contribution from TFAP2C, which is critical for specifying TE.^47^^,^^48^^,^^54^^,^^69^^,^^70^^,^^71^

The strong contribution of TFAP2C suggests that Hippo signaling is influenced by cell-type-specific TFs as hypothesized. Interestingly though, the model did not assign all cell-type-specific TF motifs the same importance (Figure 1G). For example, CDX2 and GATA3 were not predicted to help YAP1 bind, although they are critical for trophoblast identity.^50^^,^^51^^,^^52^ This suggests that the rules by which TFs boost the binding of signaling effectors are not obvious, but that, with the help of TF binding data, these rules can be learned with a deep learning model.

### YAP1 binding correlates with markers of enhancer activity

Having analyzed TSC-specific YAP1 binding, we investigated whether high YAP1 binding levels are indicative of enhancer activation. We expect YAP1 to be a strong activator based on previous molecular evidence,^72^^,^^73^^,^^74^ but many other TFs have transactivation domains, and thus it is unclear how much YAP1 contributes to enhancer activation at a genome-wide level.

Since no individual assay unambiguously measures enhancer activity,^75^ we performed experiments in TSCs to profile multiple markers of active enhancers: ChIP-nexus for RNA polymerase II (Pol II), TT-seq to capture enhancer transcription, ATAC-seq to measure chromatin accessibility, and ChIP-seq for H3K27ac found on nucleosomes flanking active enhancers^76^^,^^77^^,^^78^ (Figure S2A). We identified thousands of enhancers that showed enhancer transcription, chromatin accessibility, and H3K27ac, thus meeting our definition of being active enhancers (Table S4). We selected a few enhancers near important trophoblast genes for further characterization. Named after the nearest gene, these include a *Bmp7*, *Rin3*, *Ezr*, *Cited2*, *Amotl2*, *Bmp7*, *Dst*, and *Tjp1* enhancer. They were validated by cloning the minimal central region into a luciferase reporter assay and measuring their activity in TSCs (Figure S2B).

**Figure 2:**
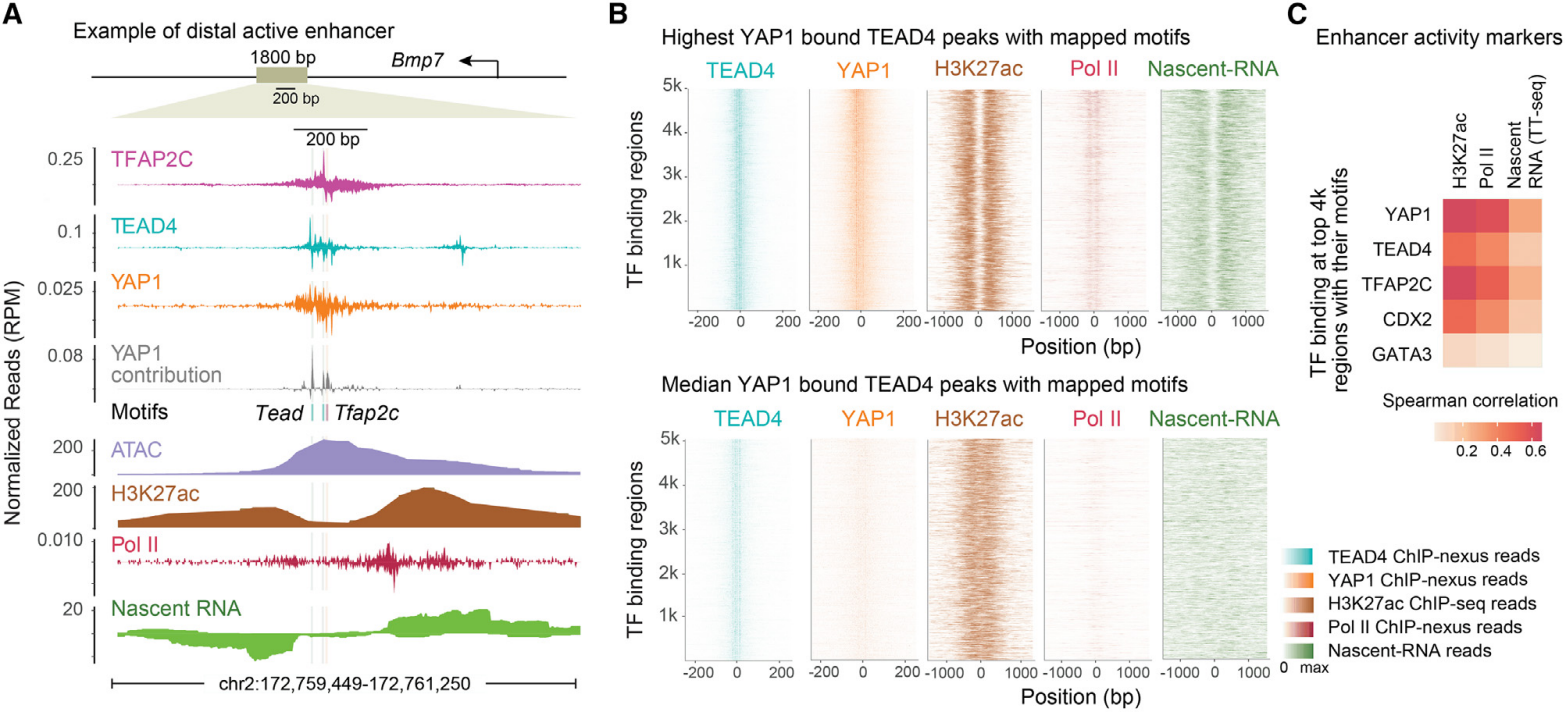
YAP1 binding correlates with enhancer activity markers (A) An example of an active enhancer ∼100 kb downstream of the *Bmp7* gene, showing ChIP-nexus TF binding for TFAP2C, TEAD4, and YAP1 alongside BPNet-mapped motifs *Tead* and *Tfap2c* and predicted YAP1 binding contribution. Additional tracks are the fragment coverage for ATAC-seq, H3K27ac ChIP-seq, Pol II ChIP-nexus data, and nascent RNA-seq derived from TT-seq. (B) Profile heatmaps of TEAD4 and YAP1 ChIP-nexus data at the 5,000 TEAD4 peaks with the highest YAP1 binding (top) and 5,000 peaks with median YAP1 binding (bottom). Regions with highest YAP1 have an active enhancer signature of H3K27ac ChIP-seq, Pol II ChIP-nexus, and Nascent-RNA reads (top). (C) A heatmap depicting Spearman correlations between ChIP-nexus TF binding and the enhancer activity markers at the top 4,000 non-promoter peaks containing their motif. YAP1 correlates best, followed by TFAP2C.

These data revealed that active enhancers are indeed associated with high levels of YAP1 binding (Figures 2A and S2E–S2H). As an example, strong binding footprints of TFAP2C, TEAD4, and YAP1 are found at the putative downstream *Bmp7* enhancer, and the contribution scores show that BPNet used the *Tfap2c* and *Tead single* motifs to predict YAP1 binding (Figure 2A). This region possesses all the characteristic features of active enhancers, with central ATAC-seq accessibility, flanking H3K27ac signal, Pol II occupancy, and bidirectional nascent RNA transcription (Figure 2A).

To examine the global correlation between YAP1 binding and the markers of enhancer activity, we selected all TEAD4 peaks with a *Tead* motif; we then compared the 5,000 regions with the highest YAP1 binding to 5,000 regions with median levels of YAP1 binding (Figure 2B). The top YAP1 bound regions showed strong H3K27ac signal, Pol II binding, and nascent transcription adjacent to the central region, while no strong evidence of enhancer activity was observed for the more lowly bound set (Figure 2B). To quantify each TF’s effect, we calculated the pairwise correlation between each TF’s binding and enhancer activity markers (H3K27ac, Pol II, and nascent RNA) (Figure 2C). Among all TFs, YAP1 binding correlated best with the enhancer activity markers. Given the strong transactivation potential of YAP1,^72^^,^^73^^,^^74^^,^^79^ we conclude that YAP1 binding is an important determinant for enhancer activation in TSCs, and hence predicting YAP1 binding should serve as a proxy for predicting enhancer activity.

### Enhancer activation involves DNA-distance-dependent TF cooperativity

If YAP1 binding occurs cooperatively and promotes enhancer activation, the cooperating motifs might activate transcription synergistically. Synergistic activation by two motifs has been documented,^16^^,^^80^^,^^81^^,^^82^ but the mechanisms are not clear and could vary. We focused on the *Tead single* and *Tfap2c* motifs since this motif pair is frequently found at active enhancers (Figure 2). In addition, genes near these active enhancers are enriched for cell fate commitment and GTPase regulation (Figure S2C), consistent with previous studies.^43^^,^^51^^,^^54^^,^^69^ Synergistic activation could occur if the *Tead single* and *Tfap2c* motif not only contribute to activation on their own but cause additional activation by promoting YAP1 binding. If so, the activity of such an enhancer would depend on BPNet-learned syntax rules for YAP1 binding and thus may depend on the distance between the motifs.

To test for synergistic activation, we performed luciferase assays using the 200 bp minimal *Bmp7* enhancer, which has a *Tead single* and *Tfap2c* motif (shown in Figure 2A). To perturb combinations of these motifs in a controlled way while reducing the chance of introducing unknown variables, we used BPNet: for each motif, we mutated the two bases that contributed most to the predictions and tested whether this led to decreased YAP1 binding (Figure S2D).

**Figure 3:**
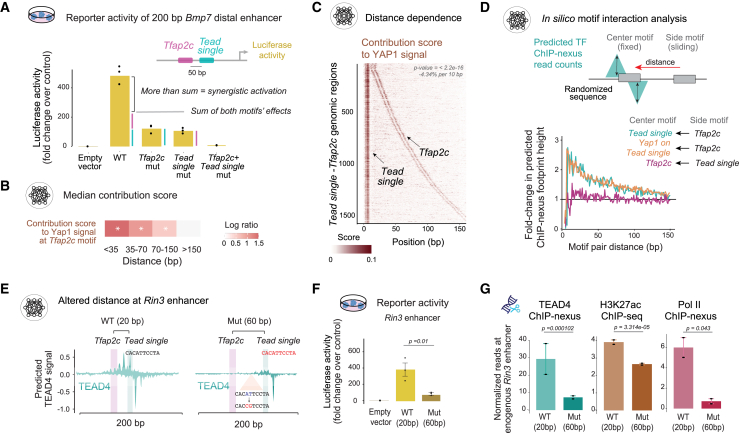
Enhancer activation involves DNA-distance-dependent cooperativity (A) Luciferase assay of a 200 bp *Bmp7* enhancer (mm10*-chr2:172*,*760*,*183-172*,*760*,*382*) in TSCs shows that the *Tead single* and *Tfap2c* motifs function synergistically, producing reporter activity greater than the sum of each individual motif. Black dots show the three biological replicates per construct. (B) The BPNet contribution scores of *Tfap2c* motifs toward YAP1 binding are significantly higher when they are in close distance to a *Tead single* motif For each distance interval, the median log ratio of the contribution scores over the baseline scores where *Tfap2c* motif >150 bp away is shown (∗*p* < 2e−16, Wilcoxon test). (C) Heatmap of YAP1 binding contribution scores at genomic regions ordered by *Tead single-Tfap2c* motif distance. The contribution of both motifs decreases with larger distances. Linear regression gives a −4.34% slope per 10 bp and *p* < 2.2e−16 (Table S5 and STAR Methods). (D) By injecting motifs *in silico* into randomized sequences, the average enhancement of TF binding to the center motif in the presence of a side motif is predicted by BPNet.^15^ The results show a distance-dependent enhancement of TEAD4 and YAP1 binding in the presence of a *Tfap2c* motif. (E) Predicted TEAD4 binding at the *Rin3* enhancer where the *Tfap2c* and *Tead single* motifs are 20 bp apart (left) and after the distance was increased to 60 bp between motifs (right). The motif was moved by inserting an identical new motif further away and mutating the most important bases within the original *Tead single* motif. (F) Luciferase assays of the wild-type (WT) and mutated (Mut) 200 bp minimal *Rin3* enhancer in TSCs, all in three biological replicates and normalized to the empty vector control, show a significant decrease (*p* < 0.05, Student’s t test). (G) After mutating the endogenous *Rin3* enhancer through sequential CRISPR (Mut), TEAD4 ChIP-nexus binding (left), H3K27ac ChIP-seq enrichment (center), and Pol II ChIP-nexus occupancy (right) were all reduced compared with WT. The scale for ChIP-nexus is reads per million (RPM), for H3K27ac levels log2(ChIP-seq/WCE reads), in a 1 kb window. Two biological replicate values (black dots); error bars show standard deviation (SD). The *p* values were derived by DEseq2 (v.1.34).^83^

Luciferase assays showed that mutating either motif alone was sufficient to strongly reduce the activity while mutating both almost completely abolished the activity (Figure 3A). Thus, in the presence of one motif, the putative *Bmp7* enhancer produced only moderate activity, while together, they resulted in activity that exceeded the sum of each motif’s effect. These results show that the *Tead single* and *Tfap2c* motifs mediate activation synergistically, presumably at least in part by increasing YAP1 binding.

To test whether the *Tfap2c* motif boosts YAP1 binding in a distance-dependent manner, we examined the contribution scores of all *Tfap2c* motifs found near a *Tead single* motif. This showed that *Tfap2c* motifs had significantly higher scores in regions where the motif was within nucleosome distance (<150 bp) and showed even higher scores when closer (Wilcoxon test p < 2e−16, Figures 3B and S3A). When we plotted these contribution scores in the genomic regions directly, they visually decreased with further distances between the *Tead single* motif and the *Tfap2c* motif (Figures 3C, S3C, and S3D). This suggests that TFAP2C enhances YAP1 binding in a distance-dependent manner through soft motif syntax.

We next investigated whether TFAP2C directly helps the recruitment of YAP1 or whether the effect on YAP1 binding is mediated through increased TEAD4 binding (Figure 3D). To distinguish between these possibilities, we performed *in silico* experiments with randomized sequences and analyzed the binding enhancement of TEAD4, YAP1, and TFAP2C with different motif distances. This revealed that YAP1 and TEAD4 binding both depend on the distance of the nearby *Tfap2c* motif, causing an over 2.5-fold increase in binding of both when the *Tfap2c* motif is close (Figures 3D and S3B). Notably, the reverse was not necessarily true: TFAP2C binding was not substantially increased (<1.5-fold) in the presence of a nearby *Tead single* motif (Figure 3D), but showed some increase in the presence of a *Tead double* motif (Figures S3B and S3D). Given that binding cooperativity is usually assumed to be mutual,^84^ this directionality is surprising but consistent with previous observations of soft motif syntax.^15^

To validate the *Tead single-Tfap2c* soft motif syntax, we performed luciferase reporter experiments on the putative *Rin3*, *Dst*, and *Adcy7* enhancers. Using BPNet predictions as a guide for designing experiments, we changed the distances between the *Tead single* and *Tfap2c* motifs at three independent regions, by deleting the *Tead single* motif through minimal mutations and introducing the same motif at a different location (Figures 3F and S3E). In all three cases, the reporter activity of the enhancer changed in the expected direction. For example, when we moved the *Tead single* motif in the minimal *Rin3* enhancer further away from the *Tfap2c* motif (from 20 bp away to 60 bp away), BPNet predicted lower TEAD4 binding (Figure 3E). This lower binding mirrored the lower activity measured in the luciferase assay (Figure 3F). Moving the two motifs closer to each other increased the luciferase reporter activity of the minimal *Dst* and *Adcy7* enhancers (Figure S3E).

To confirm that these distance effects are also observed in the genomic context, we performed CRISPR-Cas9-induced mutations in TSCs using homologous recombination on the endogenous *Rin3* enhancer (Figures S3F and S3G). ChIP experiments on this edited cell line confirmed the reduced TEAD4 binding, H3K27ac, and Pol II levels at the *Rin3* enhancer (Figure 3G), while other enhancers remained unchanged (Figure S3H). This demonstrates that changing the distance between motifs through controlled minimal mutations measurably affects enhancer activity markers in an *in vivo* endogenous context.

Taken together, we have identified genome-wide rules by which cell-type-specific TF motifs can enhance the binding of the Hippo signaling effectors. We validated that *Tfap2c* motifs enhance TEAD4/YAP1 through soft motif syntax in a distance-dependent manner, resulting in synergistic enhancer activation. This could represent a general mechanism for how cell-type-specific TFs boost the activity of signaling pathways.

### The *Tead double* motif is a canonical element of the Hippo pathway

So far, we have focused on the genome-wide rules and mechanisms by which cell-type-specific TFs influence Hippo pathway activity. To push the boundaries of what can be learned with our approach, we next asked whether we could also discover novel molecular details of the Hippo pathway effectors themselves, which are mechanistically well studied.

Notably, BPNet discovered the strictly spaced *Tead double* motif (Figure 1E), which is not considered a canonical regulatory element of the Hippo pathway,^43^^,^^85^^,^^86^ although it has been discovered multiple times.^74^^,^^87^^,^^88^ The first characterization occurred on the SV40 enhancer, but its identity remained unclear since it did not resemble the *Tead single* motif.^89^^,^^90^^,^^91^^,^^92^^,^^93^ Even after the *Tead double* motif had been discovered in *Drosophila* and cancer cells,^74^^,^^87^^,^^94^^,^^95^ it often remained unreported in genomics studies.^43^^,^^96^

**Figure 4:**
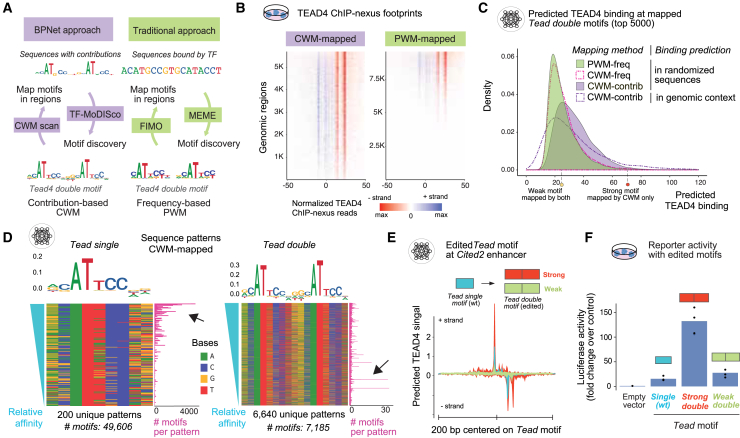
The Tead double motif is widespread, highly variable, and active (A) Comparison of the BPNet and traditional approach for *de novo* motif discovery and mapping the *Tead double* motif in genome sequences. The BPNet approach uses TEAD4 contribution scores and CWM motifs, while MEME was used on the central 101 bp of TEAD4 peaks to derive frequency-based PWMs and map motifs by match scores. With correct settings, both methods identified the *Tead double* motif. (B) However, *Tead double* motifs mapped by CWM scanning consistently show TEAD4 *in vivo* ChIP-nexus binding footprints throughout, while motifs mapped by PWM using FIMO show much fewer footprints; + strand (blue) and − strand (red). Regions were centered on the motif’s left side and sorted by ChIP-nexus signal. (C) Predicted TEAD4 signal for the top 5,000 *Tead double* motifs from each category shows that the contribution scores are key for the CWM-mapped motifs’ quality. The frequency-based PWM- or CWM-mapped motifs injected into randomized sequences perform similarly, while contribution-based CWM-mapped motifs have higher predicted values. The same CWM-mapped motifs predicted in their native genomic context show a wider distribution. (D) All unique sequence patterns for the *Tead single* and *double* motifs, sorted by relative motif affinities (teal, left) and CWM logo on top. The motif frequency (purple, right) shows that high-affinity *Tead single* motifs occur most frequently, while *Tead double* motifs do not (black arrow). (E) BPNet-predicted TEAD4 binding profile at the *Cited2* enhancer when the *Tead single* motif was replaced with a strong or a weak *Tead double* motif. (F) Luciferase assay of the 200 bp minimal *Cited2* enhancer (mm10-chr10:17,579,590-17,579,789), normalized over the empty vector control, for the three motifs, each in three biological replicates.

To test whether the *Tead double* motif has simply been overlooked or whether the BPNet approach is particularly suitable to learn this motif, we analyzed our data using traditional genomics approaches (Figure 4A). We found that it is easy to miss the *Tead double* motif by MEME or HOMER (Figure S4A), but with prior knowledge of suitable settings, a position weight matrix (PWM) similar to that discovered by BPNet/TF-MoDISco can be identified (Figure 4A). However, when FIMO was used to map motif instances in the bound genomic regions with this PWM, the mapped motifs disagreed with the *in vivo* TEAD4 ChIP-nexus data. While CWM-mapped *Tead double* motifs consistently show strong TEAD4 footprints, confirming their correct mapping, the PWM-mapped motifs show far fewer footprints (Figure 4B). This problem was less pronounced with *Tead single* motifs, which mapped more accurately with PWM scanning (Figure S4B). This shows that BPNet outperforms traditional methods in mapping functional motif instances,^15^ and that *Tead double* motifs are particularly challenging to map.

We next performed *in silico* experiments to test whether the *Tead double* motif itself was learned more accurately by BPNet or whether the genomic context provided the necessary context to make better predictions (Figure 4C). The key to CWM scanning is that it considers BPNet model-derived contribution scores in each individual genomic region (Figure 4A). These contribution scores depend on the binding strength of the motif itself and the genomic context, such as neighboring motifs. To distinguish which one was key, we *in silico* injected the PWM-mapped and CWM-mapped motifs into a randomized sequence background and predicted TEAD4 binding (Figure 4C, methods). This revealed that the CWM-mapped motifs themselves had higher predicted binding than the PWM-mapped motifs, and that this was due to the contribution scores. Genomic context modulated the binding predictions, but did not explain the better performance of CWM scanning (Figures 4C and S4C). This shows that the CWM-mapped *Tead double* motifs are more accurate because they incorporate BPNet’s learned binding strength, and that the binding strength of this motif is not well modeled by a PWM.

The challenging aspect of the *Tead double* motif is that it is a long sequence pattern where many bases do not strongly contribute and are highly variable among the mapped motif sequences (Figure 4D). When we analyzed the sequence patterns of the more regular *Tead single* motifs, the vast majority are commonly occurring patterns, with high-affinity motifs being the most frequent (Figure 4D, left). In contrast, only a tiny fraction of the mapped *Tead double* motifs have recurring sequence patterns, and the most frequent patterns are not those with the highest predicted affinity (Figure 4D, right). This can explain why a frequency-based PWM representation does not accurately reflect the binding strength and why it has been difficult to identify the *Tead double* motif on the SV40 enhancer, while the BPNet model learned this motif (Figure S4D).

Having established the widespread occurrence of the *Tead double* motif, we asked how much the *Tead double* motif promotes enhancer activity compared with a *Tead single* motif. Using our validated putative *Cited2* enhancer, we replaced the high-affinity *Tead single* motif with either a weak *Tead double* motif (mapped by both PWM and CWM scanning), and a strong *Tead double* motif (mapped by CWM scanning only, highlighted in Figure 4C). BPNet predicted that replacement with the strong *Tead double* motif caused a large increase in TEAD4 binding, while the weaker one caused a reduction in binding (Figure 4E). When assayed in a luciferase assay, the strong *Tead double* motif caused an over 8-fold increase in activity compared with the wild-type *Tead single* motif. Interestingly, even the weak *Tead double* motif showed increased activity (∼1.9-fold) over the *Tead single* motif despite lower TEAD4 binding (Figure 4F). This shows that even a weaker *Tead double* motif is highly active, explaining why it can be functional in so many different sequence patterns.

These results suggest that the *Tead double* motif is an important element of the Hippo pathway in TSCs. To test whether this can be generalized to other TEAD family members and cell types, we analyzed BPNet models trained on TEAD1-4 ChIP-seq data from the ENCODE portal^97^^,^^98^ (https://www.encodeproject.org/). BPNet discovered the *Tead double* motif in diverse human cell types for different TEAD family members (Figure S4E), showing that the *Tead double* motif is generally a widespread canonical motif of the Hippo pathway.

### Genome-wide TEAD4 cooperativity through labile protein-protein interactions

**Figure 5:**
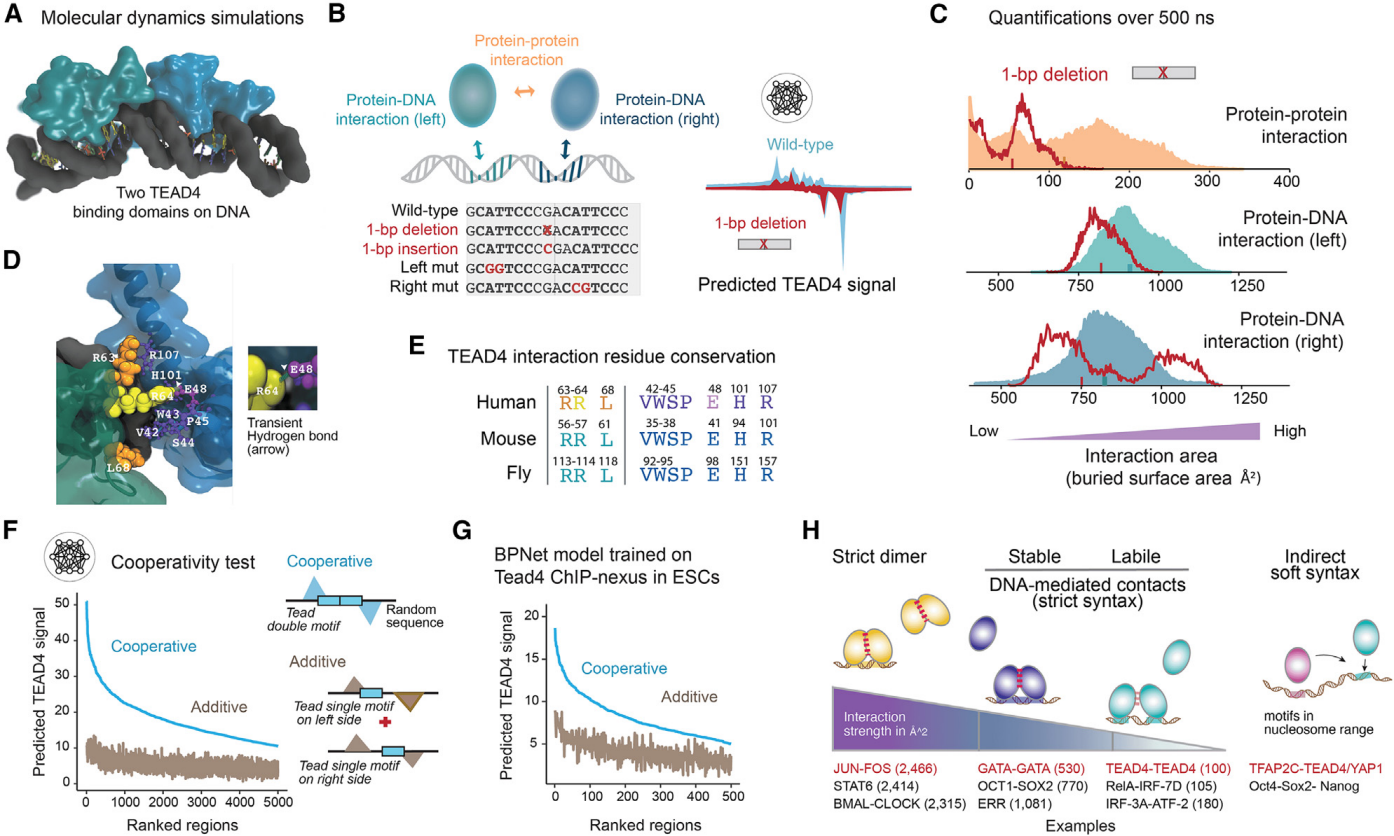
BPNet and MD simulations reveal insights into TEAD4 cooperative binding at double motifs (A) Using the known structure of human TEAD4 bound to a single motif,^102^ we constructed a model of two TEAD4 DNA binding domains simultaneously bound to a high-affinity *Tead double* motif. (B) Graphic showing which interactions were measured to quantify binding strength and which sequences were used as controls. The 1-bp deletion was chosen because BPNet predicts it to cause lower TEAD4 footprints (right), suggesting that cooperative binding depends on optimal spacing. (C) Buried surface area distributions from the MD simulations with the high-affinity *Tead double* motif (GCATTCCCGACATTCCC) shown as solid areas, and the 1-bp deletion (GCATTCCC**x**ACATTCCC) shown as a red line. Lower protein-protein interaction values for the 1-bp deletion and insertion suggest lower cooperativity at suboptimal spacing (see also Figure S5D). The mean protein-DNA interaction is not a reliable measurement for sequence affinity but the difference between the two sides is a sequence-specific feature that we experimentally validated (Figure S5N, right). (D) TEAD4 residues involved in interprotein interactions (defined by ≤4 Å in >20% of simulation frames) are shown as space-filling spheres on the left TEAD4 and as ball-and-stick atoms on the right TEAD4. In a representative frame, R64 and E48 form a hydrogen bond (green dashed line, inset), but it forms/dissociates throughout the trajectory. A simulation video shows the entire protein-protein interaction dynamics (Video S1). (E) Interactions residues are conserved across *Drosophila* Scalloped, mouse TEAD4, and human TEAD4 based on Clustal Omega multiple sequence alignment (UniProt IDs: P30052, Q62296, Q15561). (F) *In silico* analysis of the TEAD4 cooperativity in mouse TSCs on all mapped *Tead double* motif sequences, injected into random sequences either as a whole (cooperative) or each half separately and then added (additive). The predicted signal was summed in a 50 bp window and averaged across all random sequences. Motifs were ordered by the predicted TEAD4 signal on the whole motif. (G) Same as in (F), but mapped *Tead double* motifs and predictions were in mouse ESCs. (H) Quantification of protein-protein buried surface area (Å^2^) from structures of TFs that form dimers in solution (PDB: 1JNM [AP-1]; 4Y5W [STAT6]; 4H10 [BMAL:CLOCK]), TFs that have stable DNA-mediated interactions (PDB: 3DFV [GATA-GATA]; 104X [OCT1-SOX2]; 8CEF [ERR]), TFs with weak interactions (our simulations of TEAD4, PDB: 5GZB; RelA-IRF-7D and IRF-3A-ATF-2^103^), and TFs that cooperate with soft syntax and may not directly interact; examples include TFAP2C and TEAD4, and OCT4-SOX2 and NANOG.^15^ Highlighted in red are TFs for which motifs were identified here with BPNet.

The nature of the *Tead double* motif, its strong activity, and strict spacing, suggest that it is bound cooperatively by two TEAD4 molecules. Such cooperativity is also supported by previous gel shift analyses,^87^^,^^94^^,^^95^^,^^90^^,^^99^ but it is not known how strong the two TEAD4 molecules interact with each other. They could already come as a dimer, as known for JUN-FOS,^100^ or they could only interact on DNA when a corresponding DNA sequence brings them into contact.^24^^,^^101^ We, therefore, explored the mechanism of TEAD4 cooperativity using BPNet and all-atom MD simulations (Figure 5).

The MD simulations were performed by placing two TEAD4 DNA binding domains (PDB: 5GZB)^102^ on the high-affinity functional *Tead double* motif from the putative *Tjp1* enhancer (Figures 5A, 5B, S5A, and S5B). This complex was highly similar to one predicted by AlphaFold3^104^ (Figure S5M) and remained stable during the simulations over 500 ns, allowing us to quantify the protein-DNA and protein-protein interactions over time (measured as buried surface area Å^2^) (Figures 5C and S5G, left). We also confirmed that the results were similar using the *Tead double* motif from the putative *Amotl2* enhancer (Figures S5A and S5B, bottom, and S5E) or when using a different force field (CHARMM27 instead of FF19SB)^105^ (Figure S5F).

Our MD simulations revealed that the intermolecular TEAD4 protein-protein interactions are sensitive to the DNA template, consistent with BPNet’s predicted changes in binding levels (Figures S5H and S5N, left). The protein-protein interactions were strongest in the presence of the correctly spaced *Tead double* motif, and weaker when simulating with a 1-bp deletion, a 1-bp insertion, or mutations in one-half of the DNA sequence (Figures 5B, 5C, S5C, S5D, and S5G, right). The protein-protein contacts occur through amino acid residues within a region that is important for TEAD4’s cooperative binding to the *Tead double* motif^99^ and is highly conserved between TEAD family members and across evolution (Figures 5D and 5E), consistent with the widespread role of the *Tead double* motif.

At the same time, these protein-protein interactions were surprisingly dynamic and labile (Figure 5C), arguing against TEAD4 forming dimers before binding to DNA. They changed on the scale of ∼100 ns, which is much shorter than the tens of microseconds seen for stable protein-protein complexes.^106^ The contacts made by the involved residues also varied in their molecular details over time. Contacts such as hydrogen bonds (Figure 5D, inset) formed and then dissociated, with no single interaction persisting for the entire trajectory (Video S1). This suggests that the initial DNA-templated protein-protein interactions between two TEAD4 molecules are labile and dynamic in nature, although binding of this complex *in vivo* may be further stabilized by the Hippo effector partners YAP1 or TAZ.^107^

Video S1. Labile protein-protein interaction on DNA movie.mp4

To analyze TEAD4 binding cooperativity *in vivo* and test its dependence on Hippo signaling, we leveraged BPNet (Figures 5F and 5G). Since BPNet accurately predicts TEAD4 binding on *Tead single* and *Tead double* motifs, we measured the cooperativity on the *Tead double* motif as the observed increase in binding over the expected additive binding on each *Tead* motif contained in the *Tead double* motif (Figure 5F). This showed consistently strong cooperativity across the genome, with an average of ∼4-fold higher TEAD4 binding than expected from an additive model (Figure 5F).

To test whether this cooperativity was dependent on Hippo signaling, we performed TEAD4 ChIP-nexus in mouse embryonic stem cells (ESCs) where YAP1/TAZ is not nuclear,^41^ and trained an independent BPNet model on these data (Figure S5I). In these cells, TEAD4 binding was overall lower than in TSCs, resulting in much fewer bound instances (Figures 5G, S5J, and S5K). This is consistent with TEAD4 binding being stabilized at the *Tead double* motif by partners in TSCs.^107^ However, we observed cooperativity even in ESCs, with a >2-fold increase over the additive signal (Figure 5G). This is consistent with the MD model, where some degree of TEAD4 cooperativity on the *Tead double* motif is observed in the absence of YAP1/TAZ.

Finally, we investigated whether the labile interactions we observe for TEAD4-TEAD4 are within range of what has been observed for other TFs that interact on DNA (Figure 5H). Since MD simulations are rarely performed, we quantified and compared the protein-protein buried surface area between TFs from crystal structures,^24^ preferably on TFs studied here. Our high-affinity simulation gave an average buried surface area of 112 Å^2^ between the two TEAD4 molecules. For comparison, the JUN-FOS dimer is held together through much stronger protein-protein interactions (2,466 Å^2^), consistent with dimerization before binding DNA.^100^ GATA-GATA^108^ formed intermediate interactions (530 Å^2^), suggesting that the complex is DNA templated but not as labile as the TEAD4-TEAD4 complex. Finally, we found interaction strengths akin to those of TEAD4-TEAD4 (Figure 5H), most notably those of IRF with RelA (105 Å^2^), p50 (94 Å^2^), and ATF-2 (180 Å^2^) in the enhanceosome model (Figure S5L). This example is particularly relevant as the TFs have been reported to cooperate with a measurable effect on transcription.^25^^,^^82^^,^^109^ These results point toward labile interactions being a plausible mechanism for DNA-templated cooperativity.

Taken together, our results suggest that two TEAD4 molecules cooperate with each other on the *Tead double* motif through transient and labile interactions (Figure 5H). We propose that labile interactions make this type of cooperativity highly dependent on the sequence template and allow additional stabilization by partners to further fine-tune the readout.

### A redesigned enhancer shows that the *Tead double* motif increases gene activation in mouse embryos

Having shown that the *Tead double* motif mediates TEAD4 cooperative binding genome-wide, we wanted to confirm that it mediates the response to the Hippo pathway *in vivo*. We added the *Tead double* motif to an endogenous enhancer in TSCs and reintegrated these cells into mouse embryos. We selected the putative *Ezr* enhancer because it contains a *Tead single* motif, has all the hallmarks of being an active TSC enhancer (Figure S2F), and its putative target gene *Ezr* encodes an actin-associated protein that is highly expressed in TSCs.^54^^,^^110^^,^^111^ We hypothesized that replacing the *Tead single* motif with a high-affinity *Tead double* motif would increase the enhancer’s response to Hippo signaling *in vivo*.

**Figure 6:**
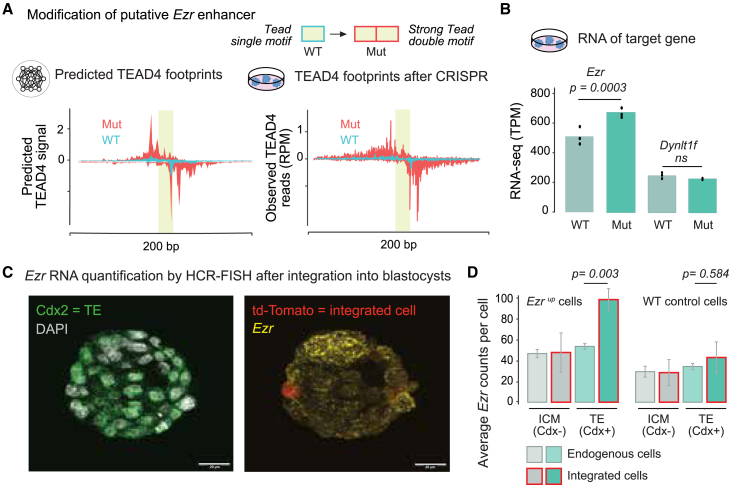
CRISPR-Cas9 enhancer design validated by BPNet increases target gene activity in mouse embryos (A) At the putative *Ezr* enhancer in mouse TSCs (mm10-chr17:6,827,705-6,827,905), the *Tead single* motif in the wild-type (WT) sequence was mutated (Mut) into a strong *Tead double* motif through CRISPR-Cas9-mediated homologous recombination. BPNet predicts increased TEAD4 binding (left); + strand on top, − strand on the same scale below, lime box: motif width. ChIP-nexus experiments confirm this change (right); scale is reads per million (RPM). (B) RNA-seq data in transcripts per million (TPM) in WT and Mut cells. Differential expression *p* values were derived using edgeR on three biological replicates. (C) HCR-FISH was performed on aggregated mouse blastocyst embryos with incorporated WT or Mut (edited Ezr^up^) cells, using probes for *Cdx2*, *Ezr*, and *td-Tomato.* (D) Quantification of average *Ezr* counts (average *Cdx2* counts shown in Figure S5E). *Ezr* transcripts were significantly increased for edited cells but not for WT cells among *Cdx2*+ TE lineage cells (Student’s t test *p* < 0.05). Error bars show standard error of the mean (SEM).

While it is typically easy to destroy the activity of an endogenous enhancer by mutating relevant TF motifs,^34^ it is more challenging to engineer mutations that increase the activity since sequence changes can have unexpected side effects.^30^^,^^31^ However, deep learning models are ideal for exploring and evaluating possible mutations, as shown in *Drosophila*.^16^^,^^112^ We, therefore, replaced the *Tead single* motif with a *Tead double* motif in such a way that BPNet predicts an increase in TEAD4 binding (Figure 6A, left).

We then used CRISPR-Cas9-induced homologous recombination to edit the endogenous enhancer in our TSCs (Figure S6A). We performed TEAD4 ChIP-nexus experiments on the edited cells and found that the TEAD4 binding footprint indeed matched the one predicted by BPNet (Figures 6A, right, and S6B). We also tested the modified enhancer sequence in the luciferase assay and found it to increase enhancer activity (Figure S6C). The increase was moderate (∼1.5-fold), likely because the wild-type enhancer activity was already high to begin with. To test whether this change affects the expression of *Ezr*, we performed RNA-seq on the edited cells with the *Tead double* motif and the wild-type cells with the *Tead single* motif. This revealed a moderate, statistically significant increase in *Ezr* transcript levels in the edited cells (Figure 6B).

We next tested whether this edit increases *Ezr* expression in mouse embryos in a cell-type-specific way. We marked the edited TSCs (and wild-type TSCs as control) with td-Tomato, aggregated these cells with early mouse embryos at the 4–8 cell stage, and analyzed the embryos when they reached the blastocyst stage, where the outer TE cells are clearly distinguishable from the inner cell mass (ICM) by their expression of *Cdx2*. We performed HCR-FISH to precisely quantify the expression of *Ezr* and *Cdx2* in these embryos (Figures 6C and S6D–S6F).

*Ezr* transcripts were specifically increased in edited cells but not wild-type cells, and only when they became TE cells with nuclear YAP1 (Figure 6C, right). Not all added TSCs maintained their TE identity but occasionally lost *Cdx2* expression and acquired ICM identity (Figure S6E), consistent with cell fate plasticity at this stage.^44^ Notably, when the edited cells lost TE identity, *Ezr* transcripts were no longer increased. These findings show that the increased activity of the *Tead double* motif is specific to the cell type with nuclear YAP1. They also demonstrate that, with the help of BPNet’s predictive framework, enhancers can be manipulated to respond more strongly to a cell-type-specific signaling pathway *in vivo*.

## Discussion

Here, we show as a proof of principle that the cell-type specificity of the Hippo signaling pathway in TSCs is encoded in the *cis*-regulatory sequences and that the sequence rules reflect the mechanisms by which these sequences are read out. Canonical effectors such as TEAD4 and YAP1 of the Hippo pathway are well studied, but how these effectors mediate different transcriptional responses in different cell types has been a challenging problem, often attributed to the combinatorial complexity of the signaling components or effects of chromatin. The fact that we can train a deep learning model to accurately predict the binding of the signaling effectors in TSCs from sequence alone, and that the binding is predictive of enhancer activation, shows that the cell-type-specific response to Hippo signaling is sequence encoded. Moreover, we found that the learned sequence rules are precise enough to reveal mechanistic insights into how signaling pathway effectors function and interact with cell-type-specific partner TFs.

The power of this approach comes from training a highly predictive base-resolution sequence model. Since the model can accurately predict test sequences it has never seen during training, it has learned general TF binding rules that apply genome-wide for the cell type it was trained on. These rules include the motif’s strength (as shown here for the *Tead double* motif) and incorporate input from neighboring motifs (e.g., the contribution of *Tfap2c* toward YAP1 binding). As a result, the tens of thousands of motifs that we mapped inside active enhancers are more accurate than motifs mapped by traditional methods. For example, a frequency-based PWM could not accurately map the long and variable *Tead double* motif in the genome, explaining why studies in the past did not identify this motif as a widespread canonical element of the Hippo pathway. Furthermore, we used the predictive accuracy of the model for designing follow-up experiments, e.g., performing minimal mutations to achieve a certain outcome.

A key focus of this study was, however, to interpret the trained model to derive mechanistic insights. We found that the model learned precise rules by which motifs combinatorially predict YAP1 binding, including syntax rules that determine how the distances between motifs affect the binding cooperativity. These syntax rules should depend on the molecular mechanisms by which the corresponding TFs interact. Since we re-discovered expected motifs and their interactions, and validated novel findings, we conclude that the sequence rules indeed mirror molecular mechanisms downstream of the Hippo signaling pathway.

The motif syntax rules suggested that cell-type-specific TFAP2C boosts YAP1 binding and enhancer activation at motif distances of up to ∼150 bp, with stronger effects at closer distances. This soft syntax, which we experimentally validated, is unlikely to involve specific protein-protein interactions and instead points to a nucleosome-mediated mechanism.^15^^,^^20^^,^^26^^,^^113^^,^^114^^,^^115^^,^^116^^,^^117^ Such a mechanism, while molecularly unclear, could explain how signaling TFs can receive input from a wide variety of TFs in different cell types without having evolved specific protein-protein interactions. Nevertheless, we found that some cell-type-specific TFs had a stronger contribution to YAP1 binding than others, suggesting that specific properties make them better partner TFs. TFAP2C is likely a strong partner because it is highly expressed, can pioneer chromatin,^118^^,^^119^ and interacts with co-activators,^120^ which could help YAP1 form condensates.^73^^,^^79^ An important challenge in the future will be to measure how TFs interact with each other in different cell types and which properties drive these interactions.

A different type of syntax was found for the *Tead double* motif, where two strictly spaced *Tead* motifs mediate cooperative TEAD4 binding. Using MD simulations, we found that this motif allows two TEAD4 molecules to directly interact with each other, but the protein-protein interactions were surprisingly labile, transient, and dependent on the DNA sequence. Such weak interactions between TFs have been observed in the enhanceosome crystal structure, but their dynamic nature and significance were not known at the time. We propose that such transient interactions are strong enough to stabilize the complex but weak enough that they depend on matching DNA sequences and can be further stabilized through signaling pathway activities.

In summary, our sequence-driven interpretable deep learning approach reveals motif dependencies and syntax rules that correspond to distinct molecular mechanisms and suggest novel hypotheses that can be further studied experimentally. For example, we also discovered a palindromic *Gata double* motif. Since previous studies have shown that GATA zinc fingers can bind DNA cooperatively,^108^^,^^121^ it should be possible to identify the structural basis for GATA3 binding to this specific motif. Importantly, our approach identifies TF cooperativities that are in many cases cell-type specific and thus open the door to systematically study how signaling pathways target different enhancers in different cell types.

### Limitations of the study

A limitation of our approach is that it depends on high-quality binding data in the cell type of interest. While BPNet can model lower-resolution ChIP-seq data,^15^^,^^122^ the cell-type specificity of signaling effectors makes obtaining comprehensive data across cell types beyond ENCODE challenging. Model training and interpretation also have limitations. For example, we noticed that YAP1 binding correlates better with enhancer activity markers than TEAD4 binding, yet the model interrogation suggested that YAP1 binding is mostly boosted through increased binding of TEAD4. Finally, the precision of MD simulations can give a deceptive sense of accuracy if the simulation does not adequately sample conformation space. Since our simulated system is large, we cannot capture a complete equilibrium ensemble, making MD results a snapshot of the possible behavior of the TEAD4-DNA complex.

## Resource availability

### Lead contact

Further information and requests for resources and reagents should be directed to and will be fulfilled by the lead contact, Julia Zeitlinger (jbz@stowers.org).

### Materials availability

CRISPR-Cas9 cell lines and complete hydrated MD trajectories generated in this study are available upon request.

### Data and code availability

• The raw and processed data for ChIP-nexus, ChIP-seq, ATAC-seq, TT-seq, and RNA-seq experiments have been deposited in GEO under series accession number GSE252463. The genomic datasets used in the paper can be viewed on the UCSC Genome Browser: Link
• The ChIP-nexus protocol description can be found at https://research.stowers.org/zeitlingerlab/protocols.html. The trained BPNet model is available at Zenodo https://zenodo.org/records/14894986. Original data, including MD simulation trajectories and microscopy images, can be accessed from the Stowers Original Data Repository at http://www.stowers.org/research/publications/libpb-2440. All code used to process and analyze the data in this paper can be accessed at https://github.com/zeitlingerlab/Dalal_hippo_signaling_2024.

## Acknowledgments

We thank Žiga Avsec, Robb Krumlauf, Helen McNeill, Anshul Kundaje, and Zeitlinger lab members for their helpful comments and suggestions on the manuscript. We thank the following Stowers Institute core facilities for their support: Sequencing and Discovery Genomics (Anoja Perera, Michael Peterson, and Amanda Lawlor), Histology (Dai Tsuchiya, Yongfu Wang, and Seth Malloy), Transgenic and Reproductive Technologies team (Michael Durnin and Andrea Moran), Cells Tissues and Organoids Center (Yan Wang, Naresh Kumar Rajendran, Sonia Ghosh, Maria Katt, Olga Kenzior, Shilpa Waduwawara, and Chongbei Zhao), Lab Services (Stacey Walker), Cytometry (Kevin Ferro, Jose Javier, KyeongMin Bae, and Jeff Haug), and Computational Biology (Hua Li, Madelaine Gogol, and Hassan Huzaifa). The research reported in this publication was supported by the 10.13039/100007795Stowers Institute for Medical Research, United States and NIH grant R01HG010211 to J.Z.

## Author contributions

K.D. and J.Z. conceived the project as part of K.D.’s thesis research to fulfill the University of Kansas Medical Center requirements. K.D. and J.Z. designed the genomics and other experiments that K.D. performed. Deep learning model training, computational analysis, and *in silico* experiments were performed by K.D. and M.W. Additional genomics data analyses were done by K.D. ATAC-seq experiments were performed by S.K. MD experiments were conceived and designed by C.M., K.D, and J.Z.; performed by C.M.; and analyzed by C.M., K.D., and J.Z. Embryo aggregation, imaging, and analysis were conceived by K.D. and J.Z. Imaging and analysis was performed by M.C.M. The manuscript was prepared by K.D. and J.Z. with input from all authors.

## Declaration of interests

J.Z. owns a patent on ChIP-nexus (no. 10287628).

## STAR★Methods

### Key resources table

**Table:** 

REAGENT or RESOURCE	SOURCE	IDENTIFIER
**Antibodies**		

Mouse monoclonal anti-TEAD4	Abcam	ab58310
Goat polyclonal anti-TFAP2C	R&Dsystems	AF5059
Rabbit polyclonal anti-CDX2	Bethyl Laboratories	A300-692A
Rabbit monoclonal anti-GATA3	Cell Signaling	5852T
Rabbit polyclonal anti-YAP1	Cell Signaling	14074S
Rabbit monoclonal anti-Pol II	Cell Signaling	D8L4Y
Rabbit polyclonal anti-H3K27ac	Active Motif	39135
Mouse monoclonal anti-CDX2 for immunofluorescence	BioGenex	MU392A-5UC
Rabbit monoclonal anti-Nanog for immunofluorescence	Cell Signaling	8822
Anti-rabbit IgG Alexa Fluor 488 secondary antibody	Biotium	20015
Anti-rabbit IgG Alexa Fluor 647 secondary antibody	Biotium	20047

**Chemicals, peptides, and recombinant proteins**		

37% formaldehyde solution	VWR	50-00-0
Dynabeads Protein A	ThermoFisher	10008D
phi29 DNA polymerase	New England Biolabs	M0269S
Lambda exonuclease	New England Biolabs	M0262S
Q5 High-Fidelity 2x Master Mix	New England Biolabs	M0492S
dNTP solution mix	New England Biolabs	N0447S
RNase A	ThermoFisher	EN0531
Phenol:chloroform:isoamyl alcohol (25:24:1) (v/v/v)	VWR	136112-00-0
Proteinase K	ThermoFisher	25530049
DAPI	BioLegend	422801
crRNA and ssODN sequences	IDT	Table S2
Alt-R HiFi Cas9 Nuclease V3 protein and tracerRNA, ATTO-550	IDT	1081059 and 1075928
rLV.EF1.tdTomato-9	Takara	0036VCT

**Critical commercial assays**		

End Repair Module	New England Biolabs	E6050S
dA-Tailing Module	New England Biolabs	E6053S
Quick Ligation Kit	New England Biolabs	M2200S
Monarch DNA Gel Extraction Kit	New England Biolabs	T1020
Monarch PCR & DNA Cleanup Kit	New England Biolabs	T1030
Hybridization Chain Reaction (HCR) v3.0	Molecular Instruments	N/A
Infusion cloning	Takara	638947
Dual-Glo luciferase assay system	Promega	N1521
TruSeq Stranded Total RNA Library Prep Kit with Ribo-Zero Gold Set	Illumina	RS-122-2303
TruSeq poly-A Stranded mRNA Library Prep Kit	Illumina	20020595
NEBNext Ultra II DNA library prep kit	NEB	E7645

**Deposited data**		

Raw and analyzed NGS and PBM data	This paper	GEO: GSE252463
Trained deep learning model	This paper	https://zenodo.org/records/14894986
Raw images and MD trajectories	This paper	https://collaboration.stowers.org/d1e17b38-9a3d-4900-a623-ac32836b32fc/

**Experimental models: Organisms/strains**		

Mouse Trophoblast Stem Cells (TSCs)	Singh et al.^55^	https://pubmed.ncbi.nlm.nih.gov/33458704/
Mouse Embryonic Stem Cells (ESCs)	Avsec et al.^15^	https://www.nature.com/articles/s41588-021-00782-6#Sec10
First CRISPR TSC line: CRISPR-Cas9 at *Tead motif* within locus chr17:6,827,802-6,827,811 in mouse Trophoblast Stem Cells (TSC)	This paper	N/A
Second CRISPR TSC line: CRISPR-Cas9 at *Tead motif* within locus chr12:102,262,024–102,262,033 in mouse Trophoblast Stem Cells (TSC)	This paper	N/A

**Oligonucleotides**		

Oligonucleotides for ChIP-nexus, see Table S2	IDT	https://research.stowers.org/zeitlingerlab/protocols.html
Illumina Index primer 1: 5′-CAAGCAGAAGA CGGCATACGAGAT[i7]GTCTCGTGGGCTCGG-3′	IDT	https://support-docs.illumina.com/SHARE/AdapterSequences/Content/SHARE/AdapterSeq/Nextera/SequencesNextera_Illumina.htm
Illumina Index primer 2: 5′-AATGATACGGC GACCACCGAGATCTACAC[i5]TCGTCGGCAGCGTC-3′	IDT	https://support-docs.illumina.com/SHARE/AdapterSequences/Content/SHARE/AdapterSeq/Nextera/SequencesNextera_Illumina.htm
Illumina Transposase adapter read 1 (Nextera A): 5′- TCGTCGGCAGCGTCAGATGTGTATAAGAGACAG-3′	IDT	https://support-docs.illumina.com/SHARE/AdapterSequences/Content/SHARE/AdapterSeq/Nextera/SequencesNextera_Illumina.htm
Illumina Transposase adapter read 2 (Nextera B): 5′- GTCTCGTGGGCTCGGAGATGTGTATAAGAGACAG-3′	IDT	https://support-docs.illumina.com/SHARE/AdapterSequences/Content/SHARE/AdapterSeq/Nextera/SequencesNextera_Illumina.htm
Mosaic end primer:/5Phos/CTGTCTCTTATACA/3ddC/	IDT	Tn5mC1.1-A1block

**Recombinant DNA**		

pETM11-Sumo3-Tn5 plasmid	Hennig et al.^123^	E54K,L372P
His6-tagged SenP2 protease plasmid	Hennig et al.^123^	N/A

**Software and algorithms**		

FIJI	Schindelin et al.^124^	https://fiji.sc/
Cutadapt v.2.5	Martin 2011^125^	https://cutadapt.readthedocs.io/en/v2.5/
Bowtie (v.1.1.2) and Bowtie2 (v.2.4.2)	Langmead and Salzberg^126^	https://bowtie-bio.sourceforge.net/bowtie2/manual.shtml
MACS2 v.2.2.6	Zhang et al.^127^	https://github.com/macs3-project/MACS
Irreproducible Discovery Rate framework v.2.0.3	Li et al.^128^	https://github.com/nboley/idr
STAR v.2.7.3	Dobin et al.^129^	https://code.google.com/archive/p/rna-star/
deepTools2 v.3.1.3	Ramírez et al.^130^	https://deeptools.readthedocs.io/en/latest/
BPNet software v.0.0.23	Avsec et al.^15^	https://github.com/kundajelab/bpnet/
Keras v.2.2.4	Chollet F^131^	https://pypi.org/project/keras/
TensorFlow1 backend v.1.7	Abadi et al.^132^	https://www.tensorflow.org/install/pip
Adam optimizer	Kingma and Ba^133^	N/A
DeepLIFT v.0.6.9.0	Shrikumar et al.^63^	https://github.com/kundajelab/DeepExplain
TF-MoDISco v.0.4.2.2	Shrikumar et al.^64^	https://github.com/kundajelab/tfmodisco
DESeq2 v.1.34.0	Love et al.^83^	https://bioconductor.org/packages/release/bioc/html/DESeq2.html
R v.4.1.1 and v.4.2.3	R core team	https://www.r-project.org/
Rstudio	RStudio	https://rstudio.com
ggplot2 v.3.5.1	Wickham^134^	https://ggplot2.tidyverse.org/
VMD 1.9.3	Humphrey et al.^135^	http://www.ks.uiuc.edu/Research/vmd/
AmberTools20 and CHARMM27	Case et al.^136^ and MacKerell et al.^104^	https://ambermd.org/AmberTools.php https://mackerell.umaryland.edu/charmm_ff.shtml#gromacs
MEME v.5.3 and v.5.5.3	Bailey et al.^137^	https://meme-suite.org/meme/tools/meme
FIMO v.5.5.3	Grant et al.^138^	https://meme-suite.org/meme/tools/fimo
AlphaFold3	Abramson et al.^102^	https://alphafoldserver.com/
Napari v0.4.0	Ahlers et al.^139^	https://doi.org/10.5281/zenodo.3555620
Cellpose deep learning	Stringer et al.^140^	www.cellpose.org

**Other**		

All code and analyses that contributed to this work	This paper	https://github.com/zeitlingerlab/Dalal_hippo_signaling_2024
Bioruptor Pico sonication device	Diagenode	https://www.diagenode.com/en/p/bioruptor-pico-sonication-device
Confocal scanning microscope	Zeiss	800
Spinning disk microscope	Nikon	Eclipse Ti2

**Quantification and Statistical Analysis**		

Linear regression model was implemented in R v.4.1.1 using the lm function.From the fitted model,key parameters (slope, intercept, *p*-value) were extracted. (Table S5)	R core team	https://www.r-project.org/

### Method details

#### Mouse stem cell culture

Mouse trophoblast stem cells (TSCs) were a gift from Vijay Pratap Singh and were maintained in a feeder-free culture as described.^55^ Briefly, feeder conditioned medium (Feeder-CM) was prepared by culturing γ-irradiated MEFs (mouse embryonic fibroblasts) in TS medium (RPMI 1640 medium, FBS 20%, 50 μg/mL of Penicillin and streptomycin (100×), 1 mM of Sodium pyruvate (100 mM), 0.1 mM of β-Mercaptoethanol (20 mM), 2 mM GlutaMAX (200 mM) for 72 h and then filtered with a 0.45 μm filter. 70% Feeder-CM plus 30% TS medium (70cond) supplemented with growth factor FGF4 (R&D System) and heparin (Sigma) (70cond +1.5x F4H Medium) was used to maintain TSCs in feeder-free conditions. Mouse embryonic stem cells (ESCs) were cultured and maintained as previously described.^15^

#### ChIP-nexus, PAtCh-Cap, and ChIP–seq experiments

For each ChIP-nexus experiment, 10^6^ TSCs were used. Cells were washed with PBS and cross-linked with 1% formaldehyde (Fisher Scientific) in PBS for 10 min at RT. The reaction was quenched with 125 mM glycine. Fixed cells were washed twice with cold PBS and resuspended in cold lysis buffer (15 mM HEPES pH 7.5, 140 mM NaCl, 1 mM EDTA, 0.5 mM EGTA, 1% Triton X-100, 0.5% *N*-lauroylsarcosine, 0.1% sodium deoxycholate and 0.1% SDS), incubated for 10 min on ice and sonicated with a Bioruptor Pico (Diagenode) for five cycles of 30 s on and 30 s off. The ChIP-nexus procedure and data processing were performed as previously described,^38^ except that the ChIP-nexus adapter mix contained four fixed barcodes (ACTG, CTGA, GACT, and TGAC), and PCR library amplification was performed directly after circularization of the purified DNA fragments (without the addition of the oligo and BamHI digestion). PAtCh-Cap was performed as previously described^141^ with 10% of sheared chromatin from 10-^6^ TSCs. ChIP-seq experiments, including a whole cell extract (WCE) control, were performed as described^142^ with 10e^6^ TSCs per ChIP. For each ChIP, 5–10 μg of antibody was coupled to 50–100 μL of Protein A or Protein G Dynabeads (Invitrogen). The following antibodies were used: anti-TFAP2C (R & D systems, no. AF5059), anti-TEAD4 (Abcam, no. ab58310), anti-CDX2 (Bethyl Laboratories, no. A300-692A), anti-GATA3 (Cell Signaling, no. 5852T), anti-YAP1 (Cell Signaling, no. 14074S), anti-Pol II (Cell Signaling, no. D8L4Y), anti-H3K27ac (ChIP-seq) (Active motif, no. 39135). For all experiments, at least two biological replicates were prepared–that is, the experiments were performed on different days, starting with cells from a different passage number. Single-end sequencing was performed on an Illumina NextSeq 500 instrument (75 cycles). The full ChIP-nexus protocol can be found on the Zeitlinger lab website at https://research.stowers.org/zeitlingerlab/protocols.html.

#### Luciferase assays

Selected genomic regions of range 175–200 bp were synthesized using GeneArt Strings DNA fragments along with restriction enzyme sites for with Kpn1 (NEB, R0142) and XhoI (NEB, R0146) to clone in pNL3.2 vector. pNL3.2 vectors (Promega) were digested and cloned using an Infusion master mix (Takara) upstream of the luciferase gene. Stellar competent cells (Takara) were used for transformation and downstream miniprep (Qiagen), following the manufacturer’s protocol. The cloned sequences were confirmed using the Sanger sequencing method. 2.5-^5^ TSCs were used to transfect a total of 500 ng DNA with lipofectamine2000 in a ratio of 1:2 (DNA to lipofectamine2000), following the manufacturer’s protocol. Cells were co-transfected with 1:100 ratio of control (pGL4.54[luc2/TK]) and reporter construct (pNL3.2[NlucP/minP]). Cells were transfected in suspension for 15–20 min and resuspended with media to grow in each well of the 24-well plate.

Luciferase assays were performed using a Dual-Glo luciferase assay system (Promega). After 24 h, cells were harvested, and the NanoDLR assay protocol was followed per the manufacturer’s instructions to take luminescence measurements with SpectraMax iD3 Plate Reader. Reporter luminescence signals were normalized according to their corresponding control luminescence signals, resulting in relative luciferase activity. Replicate luciferase assay experiments were performed independently three times (Table S3).

#### TT-seq experiments

TT-seq experiments were performed in three biological replicates on TSCs across three biological replicates, as described in^123^ https://www.protocols.io/view/transient-transcriptome-sequencing-experimental-pr-3byl42y22vo5/v1. Libraries were prepared using TruSeq Stranded Total RNA Library Prep Kit with Ribo-Zero Gold Set to degrade rRNA. Approximately 10^6^ cells were seeded in a 100 cm dish (∼80% confluency) and incubated with 500 μM of 4sU (Sigma) at 37°C, 5% CO_2_ for 15 min. The cells were then collected by adding 4.5 mL of TRIzol lysis reagent (Life Technologies Corp), incubated for 5 min on ice, flash-frozen, and stored at −80°C. In the biotinylation of the 4sU labeled RNA step, acid-phenol-chloroform (ThermoFisher) was used instead of chloroform.

#### ATAC-seq experiments

For each ATAC-seq experiment, 1-^5^ or 2^5^ TSCs were harvested, washed with PBS, and resuspended in ATAC Resuspension Buffer (RSB, 10 mM Tris-HCl pH 8.0, 10 mM NaCl, 3mM MgCl_2_) with 0.1% IGEPAL CA-630. Tn5 transposition was performed as previously described.^143^^,^^144^ Briefly, the cells were incubated for 3 min on ice in ATAC RSB supplemented with 0.1% IGEPAL CA-630, 0.1% Tween 20, and 0.01% Digitonin (Promega, G9441). The reaction was quenched with ATAC RSB with 0.1% Tween 20 and centrifugation. Tagmentation took place at 37°C and 1000 rpm for 30 min in a 50 μL reaction volume containing 10 μL of 5x Tagmentation Buffer (50 mM Tris-HCl pH 7.5, 25 mM MgCl_2_, 50% DMF), 0.5 μL 10% Tween 20, 0.5 μL 1% Digitonin, 1–2 μM assembled transposome and water. Tn5 transposase was purified in-house as previously described.^145^ Tn5 was loaded with previously reported oligonucleotides Tn5ME-A/Tn5mC1.1-A1block and Tn5ME-B/Tn5mC1.1-A1block^124^^,^^146^ by mixing equal amounts of purified Tn5 protein and annealed oligonucleotides for 30 min at RT. After tagmentation, the DNA fragments were purified using the Monarch PCR & DNA Cleanup Kit (NEB). Libraries were constructed using Illumina Nextera Dual Indexing, and qPCR was used to prevent over-amplification as described.^143^ At least three biological replicates were generated, and paired-end sequencing was performed on an Illumina NextSeq 500 instrument (2 x 75 bp).

#### RNA-seq experiments

TSCs wild-type and CRISPR-Cas9 edited cells were grown separately in wells of a 6-well plate and harvested at 80% confluency (∼2^6^ cells) using 500 μL of TRIzol reagent (Life Technologies Corp). The lysate was incubated for 5 min on ice, flash-frozen, and stored at −80°C. For RNA extraction, the lysate was quickly thawed at 65°C, cooled on ice for 5 min, and vortexed. Then, 100 μL of chloroform was added per 0.5 mL of TRIzol lysis reagent, shaken vigorously for 15 s, and incubated for 3 min. The sample was centrifuged at 4°C and 7000 x g for 25 min, and the upper colorless aqueous phase was transferred to a new tube. 250 μL of isopropanol was then added, incubated for 10 min at 4°C, and centrifuged at 4°C and 12,000 x g for 10 min. The total RNA precipitate formed a white gel-like pellet at the bottom of the tube, which was washed with 75% ethanol, air-dried for 5–10 min, and resuspended in 20 μL of RNase-free water. DNase treatment was performed using the TURBO DNase kit per the manufacturer’s instructions: adding 1 μL of TURBO DNase and 2 μL of DNase buffer to the dissolved RNA and incubating at 37°C for 30 min. To inactivate TURBO DNase, the RNA samples were extracted with phenol/chloroform (Sigma). The sample was centrifuged at 4°C and 7000 x g for 25 min, and the upper colorless aqueous phase was transferred to a new tube. 250 μL of isopropanol was added, incubated for 10 min at 4°C, and centrifuged at 4°C and 12,000 x g for 10 min. The total RNA precipitate formed a white gel-like pellet at the bottom of the tube, which was washed with 75% ethanol, air-dried for 5–10 min, and resuspended in 20 μL of RNase-free water. The samples were incubated in a water bath or heat block set at 55°C–60°C for 10–15 min. The RNA concentration was determined using a NanoDrop spectrophotometer, and the RNA integrity was checked on a 2100 Bioanalyzer using an Agilent RNA 6000 Nano Kit. mRNA-stranded libraries were prepared using a TruSeq poly-A Stranded mRNA Library Prep Kit and sequenced on an Illumina NextSeq 2000 P2 platform with 2 x 100 bp single-end reads. Three biological replicates were performed for wild-type and CRISPR-Cas9 edited cells.

#### CRISPR-Cas9 experiments

In the first CRISPR TSC line, the *Tead double* motif on chr17:6,827,802-6,827,811 (mm10) was mutated from ACATTCCAGA (wild-type) to GCATTCCAGGAATTCCA (mutant). In a second CRISPR TSC line, the *Tead single* motif (CACATTCCTA) on chr12:102,262,024–102,262,033 (mm10) was first inserted at 60 bp downstream of the *TFAP2C* motif (GGGCCCCAGGGCC) and then in a second round of CRISPR-Cas9 editing *Tead single* motif was mutated from CACATTCCTA (wild-type) to CACCGTCCTA (mutant) at its original position. crRNA target sites were designed using the IDT target predictor tool by evaluating the predicted on-target efficiency score and off-target potential. Alt-R CRISPR-Cas9 crRNA was designed to contain ∼40 bases of homology from the targeted cut site (gRNA and ssODN sequences are shown in Table S2. Equimolar amounts (stock of 100 μm) of Alt-R crRNA and tracrRNA-ATTO550 were mixed to form gRNA at a final concentration of 50 μM. The mixture was heated at 95°C for 5 min and cooled at RT. The single-stranded donor oligonucleotides (ssODN) were designed to contain ∼40 bases of homology from the targeted cut site (crRNA and ssODN sequences were designed using the IDT software tool). A ribonucleoprotein (RNP) complex was formed by combining 150 pmol of gRNA (crRNA+tracrRNA) and 125 pmol of Cas9 HiFi v3 protein (IDT) with hybridization for 20 min at RT. The RNP was combined with 100 pmol of ssODN donor and 100 pmol of electroporation enhancer v2 and delivered to 1.5e^5^ cells by Neon electroporation (1,400 V, 10 m, 3 pulses; Neon Transfection System, MPK5000, Life Technologies). Immediately after electroporation, cells were cultured in 0.5 μM Alt-R HDR enhancer V2 of 0.69 mM. After 24 h, cells were washed with PBS before FACS sorting on S6 FACSymphony. Single cells were directly sorted into 96-well plates. Cells were screened for the expected mutations through paired-end sequencing on an Illumina MiSeq instrument (250 cycles). On-target indel frequency and expected mutations were analyzed using CRIS.py.^147^ Clones with the intended homozygous mutation and sequence alignments >90% were chosen for further experiments, except for the 2nd CRISPR line, where we found one Indel and SNP within 500 bp of the original *Tead single* motif position, but these changes were predicted to be neutral by BPNet.

#### Mice strains and superovulation

C57BL/6J (B6) strain of mice were used from the Stowers Institute for Medical Research (SIMR) core production colony. Three to four-week-old females were superovulated using 5IU of PMSG (Genway Biotech), followed by 5IU HCG (Sigma Aldrich) 46–48 h later. Following HCG, females were paired with B6 males and checked for a copulatory plug the next morning, indicating successful mating. Fertilized embryos were collected from the plugged females at 1.5 dpc (2-cell stage) by flushing M2 (Millipore) through the infundibulum and out the uterine horn using a blunt needle. Embryos were then incubated overnight at 37°C under 5% CO_2_ in humidified air in 4-well culture dishes containing KSOM media (Millipore). Experiments were approved by the SIMR IACUC and were performed following the committees’ guiding principles.

#### Lentivirus transduction of fluorescent td-tomato in TSCs

Two days (48 h) before the transduction of wild-type or CRISPR-Cas9 edited (putative *Ezr* region edits) TSCs, cells were seeded at 1 X 10^5^ cells per well in triplicate with 3 mL of media per well of 6-well plate. On the day of transduction, cells were small-sized colonies of about 30–40% confluence; the old media were removed and washed once with PBS and replaced with 2 mL of media. The cells were infected with prepackaged lentiviral particles (constitutive reporter vector expressing tdTomato fluorescent protein gene driven by EF1a promoter (Takara) at MOI of 20 (Stock: 3.5-^10^ TU/ml) along with polybrene (4ug/ml) (Sigma) for 24 h before being replaced with a fresh medium. Four days after transduction, the td-Tomato-positive cells were selected using puromycin antibiotic selection (1 μg/ml) (InvivoGen) and were kept under selection until the positive colonies reached 60–80% confluence. Once cells reached 80% confluence, the positive cells were FACS sorted on S6 FACSymphony, expanded, and used for embryo aggregation experiments.

#### Aggregation assays to obtain chimeric embryos

To prepare the aggregation plates, six indentations on the bottom of the 35 × 10 mm plates were made using an aggregation needle (BLS) sterilized with 70% alcohol and added a drop of KSOM. All drops of KSOM were covered with mineral oil (Sigma). On the morning of the aggregation, the embryos (8–16 cell stage) were washed through M2 and then placed in drops of Tyrode’s solution (Sigma). After about 30 s, the zona pellucida began to dissolve. Once the zona was dissolved, the embryos were picked up and rinsed through a drop of M2 to neutralize the Tyrode’s solution, then placed in a drop of KSOM. Embryos were moved from this dish to the aggregate plates, placing an embryo into each indentation. Clumps of td-Tomato transduced TSCs were then picked up with a mouth pipette and moved onto each embryo in the aggregate plate. Once settled and in contact with the embryo, the aggregation plates were cultured in an incubator at 37°C under 5% CO_2_ in humidified air for 46–48 h until the embryos reached the blastocyst stage. Chimeric blastocysts were fixed with 4% paraformaldehyde (ThermoFisher) for 20 min and washed three times with PBS before mounting them on a glass bottom plate (Cellvis) coated with poly-L-lysine (Sigma). Embryos were imaged with a Zeiss LSM800, an upright confocal laser scanning microscope.

#### Immunofluorescence stainings of chimeric embryos

A few fixed chimeric blastocysts were used for immunofluorescence stainings. The embryos were washed three times with PBS-T (PBS with 0.1% Triton X-100) and incubated in PBS-T for 1 h or longer at 4°C. Embryos were then washed two times with PBS for 10 min each and incubated with 300 μL of superblock solution (ThermoFisher) for 90 min at RT, before adding the primary antibodies: CDX2 (BioGenex-MU392A-5UC) and Nanog (Cell Signaling, 8822S) with 10 μg/mL of DAPI from BioLegend:422801. The CDX2 antibody came with a signal enhancing reagent, which was used to replace 75% of the superblock solution while incubating with the primary antibody. The embryos were incubated overnight at 4°C, covered from light. The next day, the samples were washed for 10 min each three times with PBST (0.1% Triton X-100) at RT and once with PBS at RT. Secondary antibodies were added (biotium:20015,20047) in special PBS (ThermoFisher) at a 1:300 dilution with DAPI 2 μL in 1 mL of 10 μg/mL (BioLegend) and kept on light rotation for 2 h at RT. Samples were then washed three times with PBS for 10 min. Samples were imaged immediately or kept for up to a week at 4°C before imaging. Imaging was performed with an upright confocal laser scanning microscope (Zeiss LSM800) with 40x magnification. Maximum intensity Z projections and adjustments to the brightness and contrast were performed in ImageJ/FIJI.^148^ Samples larger than the field of view were taken as tiled images and stitched with the Grid/Collection Stitching plugin in ImageJ.

#### HCR-FISH on chimeric embryos

Embryos were fixed in 4% paraformaldehyde for 20 min and washed three times in PBS +0.2% Triton X- for 10 min each. RNA FISH experiments were performed using HCR v3.0 (Molecular Instrument Inc.). The RNA sequences that were used to design probes are listed along with the chosen amplifiers and probe set size: Ezr (NM_009510.2, B4,32), CDX2 (NM_007673.3, B1,29), tdTomato (B5,16). The following amplifiers with Alexa fluorophore were used: 488, 546, and 647. The fixed embryos were serially dehydrated into methanol and stored at −20°C until use. To rehydrate the embryos, they were washed in PBS +0.1% Triton X-(PBST). Embryos were incubated in the hybridization buffer for 30 min at 37°C, then in the hybridization buffer containing the probes at 37°C for 16 h. Embryos were washed 5 times with the wash buffer for 5 min each, then 2 times in 5x SSCT (5x SSC +0.1% Tween 20). Amplifiers were snap-cooled by heating at 95°C for 90 s and cooled to RT for 30 min under dark conditions. Embryos were incubated in an amplification buffer for 30 min at RT before adding the amplifiers and incubating the embryos for 80 min at RT in a humid chamber under dark conditions. Embryos were washed 4 times in 5x SSCT for 5 min each, stained with DAPI (10 μg/ml) from BioLegend in 5x SSCT for 30 min, then washed two times in 5x SSCT. Embryos were stored in 5x SSC at 4°C until imaging. Images of labeled chimeric blastocysts were acquired with an Orca Flash 4.0 sCMOS at full resolution on a Nikon Eclipse Ti2 microscope equipped with a Yokagawa CSU W1 Spinning Disk Confocal with 50 μm pinholes. A Nikon 40x long working distance water immersion objective, NA 1.15, was used to acquire all channels with exposure times of DAPI: 20ms, Alexa 488: 200ms, Alexa 546: 250ms, and Alexa 647: 250ms.

#### Molecular dynamics simulations

System preparation and simulation procedure: Canonical B-form DNA was created for each simulated sequence using Avogadro 1.2.0.^149^ The TEAD4 structure was taken from PDB:5GZB^102^, and selenomethionine residues were replaced with regular methionine by simply renaming the selenium atom to sulfur. In order to align the protein structure from 5GZB onto the created DNA structures, we aligned the phosphorus atoms from the 4th to 10th residue on chain B of the PDB (which correspond to the bases CATTCCT) to the corresponding atoms on the created DNA. Since we simulated TEAD4 dimers, we performed this alignment twice, once for each binding site. This alignment was accomplished using VMD 1.9.3.^135^ We combined the two translated copies of the TEAD4 protein and the synthetic DNA sequence into one system using AmberTools20.^136^ We used the ff19SB force field for protein atoms,^150^ the bsc1 force field for DNA,^151^ and the OPC for water and ions.^152^ (For a control simulation using CHARMM27, the same procedure was used to create the initial structure, but the system was built in VMD and TIP3P was used as the water model.) Systems were solvated in truncated octahedra of water with a 12 Å padding between the solute and cell edge. Systems were charge-neutralized with K+ ions, and additional K+ and Cl-ions were added to bring the system to a concentration of approximately 150 mM KCl. Systems were minimized using NAMD 2.13.^125^ During minimization, a cutoff distance of 9 Å was used (12 Å for CHARMM27), and solvent bonds were held rigid, though all solute bonds were unrestrained. A timestep of 2 fs was used, and PME electrostatics was applied with a grid spacing of 1 Å. Ten thousand steps of minimization were performed. For thermalization and production, we used a GPU-enabled build of NAMD 2.14.^125^ The same parameters were used as in the minimization, except for the introduction of a Langevin piston to maintain the system pressure at 1 atm and a harmonic collective variable restraint^126^ to prevent the ends of the DNA from fraying during the simulation. This restraint was applied between H1 from the terminal guanine and N3 of the terminal cytosine. (The DNA ends with a GC pair on each end, and both ends of the DNA were restrained in the same way.) A force constant of 1 kcal/mol/Å^2^ was applied to maintain this distance at 2 Å. During thermalization, all velocities were started from zero and gradually warmed by applying a Langevin thermostat to raise the system temperature to 310 K during a ten ps simulation. The thermalized systems were equilibrated for ten ns, the only difference in configuration from the thermalization simulation being the timestep (increased from 1 fs to 2 fs) and the use of rigid bonds (all bonds, including hydrogen, was made rigid during equilibration and production. Coordinates were saved for every ps for both equilibration and production runs. Simulation stability was verified by plotting protein and DNA RMSD values; all simulations were stable. To verify that the simulations had reached equilibrium, we measured BSA values in the first and last fifth of the trajectory; they showed similar distributions in each case except for the deletion, where the right-hand TEAD4 detached from the DNA toward the end of the simulation. We have provided dehydrated trajectories along with all analysis scripts in Python (Python.org), D (dlang.org), and VMD^135^ in one folder. Complete, hydrated trajectories, totaling approximately 5 TB of data, are available upon reasonable request. All MD simulation trajectories can be accessed at https://collaboration.stowers.org/d1e17b38-9a3d-4900-a623-ac32836b32fc/.

#### ChIP-nexus data processing

ChIP-nexus and PAtCh–Cap single-end sequencing reads were pre-processed by trimming off fixed and random barcodes and reassigning them to FASTQ read names. ChIP-nexus adapter fragments were trimmed from the 3′ end of the fragments using cutadapt (v.2.5).^127^ ChIP-nexus and PAtCh–Cap reads were aligned using bowtie (v.1.1.2)^128^ and its bowtie to the *Mus Musculus* genome assembly mm10. Aligned ChIP-nexus and PAtCh–Cap BAM files were deduplicated based on unique fragment coordinates and barcode assignments. ChIP-nexus coverage was normalized was acquired through reads per million (RPM) normalization, where the ChIP-nexus sample coverage was scaled by the total number of reads divided by 10^6^. ChIP-nexus peaks were mapped using MACS2(v.2.2.6)^130^ with parameters designed to restimulate the full fragment length coverage instead of the single stop base coverage (--keep-dup = all -f = BAM --shift = −75 --extsize = 150). ChIP-nexus peaks were filtered for reproducibility in a pairwise fashion using the Irreproducible Discovery Rate framework (IDR) (v.2.0.3).^153^ The IDR framework selected the peaks used for downstream analysis from the largest pairwise comparison.

#### ChIP-seq data processing

ChIP-seq single-end sequencing reads were aligned to the *Mus Musculus* genome assembly mm10 using bowtie2 (v.2.4.2).^128^ Aligned ChIP-seq BAM files were deduplicated based on unique fragment coordinates and fragments extended based on the average experiment fragment length as determined with an Agilent 2100 Bioanalyzer. Normalized ChIP-seq coverage was acquired using the deepTools subfeature bamCompare (v.3.1.3)^154^ using parameters to generate RPKM or log_2_ fold-change scaling (--scaleFactorsMethod = None --normalizeUsingRPKM --binSize = 50) or (--scaleFactorsMethod = readCount --operation = log2 --binSize = 50). ChIP-seq peaks were mapped using MACS2 (v.2.2.6)^130^ with default parameters and an applied background coverage using the associated WCE ChIP-seq control experiment. ChIP-seq peaks were filtered for pairwise reproducibility using the Irreproducible Discovery Rate framework (IDR) (v.2.0.3).^153^

#### TT-seq data processing

TT-seq 75 bp paired-end sequencing reads were aligned using STAR(v.2.7.3)^129^ to the *Mus Musculus* genome assembly mm10 with the following parameters: outFilterMismatchNmax 2, outFilterMultimapScoreRange 0. SAMtools (v.1.14)^155^ were then used to keep alignments with mapping quality greater than 255 (-q 255), and only proper pairs (-f 2) were selected. Strand-specific BAM files for each replicate and combined were generated using the following parameters (samtools view -b -f 128 -F 16; -b -f 80; -b -f 144; -b -f 64 -F 16) and (samtools merge plus_128.bam with plus_80.bam and minus_144.bam with minus_64.bam). Normalized TT-seq coverage was generated using bamCoverage (v.3.1.3)^156^ parameter Reads Per Kilobase per Million mapped reads (RPKM).

#### ATAC-seq data processing

ATAC-seq paired-end sequencing reads were aligned using bowtie2 (v.2.4.2)^128^ to the *Mus Musculus* genome assembly mm10. Normalized ATAC-seq coverage was acquired through RPKM normalization along with following parameters: -bs = 50 --minFragmentLength 10 --maxFragmentLength 1000 --ignoreDuplicate --extendReads

#### RNA-seq data processing

RNA-seq 100 bp single-end sequencing reads were aligned to the *Mus Musculus* genome assembly mm10 using STAR (v.2.7.3)^129^ with the following parameters: outSAMtype BAM SortedByCoordinate, outSAMprimaryFlag OneBestScore,outFilterMultimapNmax 20, outFilterMismatchNoverLmax 0.1, outFilterType BySJout,alignSJoverhangMin 8, alignSJDBoverhangMin 1,outFilterMismatchNmax 999,alignIntronMin 20,alignIntronMax 1000000,alignMatesGapMax 1000000,limitBAMsortRAM 10000000000,outSAMattributes NH HI MD AS nM, quantMode TranscriptomeSAM GeneCounts. Rsem-calculate-expression (v1.3.0)^140^ was used to generate an expression table with the following parameters: no-bam-output, estimate-rspd, strandedness reverse.

#### HCR-FISH image analysis

Images were analyzed in Python 3.9. 3D masks were created with the DAPI label using the Cellpose deep learning package.^157^ After a small Gaussian blur of width 1 × 2 × 2 pixels, Cellpose segmentation was performed with the cyto model with a diameter of 60 and minimum cell size of 10000. The trophoblast cells were segmented well with this method in 3D, but the crowded inner cell mass cells were frequently corrected by hand using Napari (https://doi.org/10.5281/zenodo.3555620). The masked DAPI was expanded by 4 pixels in the xy direction to encompass more of the cytoplasm HCR label in each cell. The small HCR puncta were found by first performing a Gaussian blur of 2 × 5 × 5 width, then a Laplace filter using Gaussian derivatives with sigma = 0.1, 0.5, 0.5. Finally, the local maximum peaks in intensity are found using the Scikit-image peak_local_max function with a threshold of 11 for the CDX2 channel and 15 for both Ezrin and tdTomato channels. The number of HCR puncta found inside each masked cell was recorded. A threshold was determined to categorize a cell as CDX2 positive (greater than 5 HCR puncta) or tdTomato positive (greater than 15 HCR puncta). The threshold for tdTomato is greater because the HCR hairpin signal is sometimes found on the outside surface of the blastocyst, forming brighter and larger puncta compared to the interior cell signal, which would cause too many cells to be categorized as tdTomato positive. All thresholds are held constant between all images of blastocysts.

#### Molecular dynamics analysis and visualization

Solvent-accessible surface areas were calculated using VMD,^135^ with a 1.5 Å radius around all atoms. The buried surface area between the two systems was calculated by subtracting the surface area of the combined system from the sum of the surface areas of each component system. Plots were generated using Matplotlib,^158^ Scipy,^159^ and NumPy^160^ with Python 3.10 (python.org). The same protocol was used to calculate the buried surface area values of crystal structures and the enhanceosome model. Figures were generated with Tachyon^125^ in VMD; trajectory frames were aligned using a frame-by-frame aligner developed previously.^161^ Secondary structures were determined using STRIDE.^138^

#### BPNet model training

BPNet (v.0.0.23) architecture and software were applied as previously described.^15^ Model inputs were 1000 bp genomic sequences centered on the ChIP-nexus peaks of TF of interest. Model outputs were the predicted counts (total reads across each region) and predicted profile (coverage signal across each region) for TFAP2C, TEAD4, CDX2, YAP1, and GATA3 ChIP-nexus experiments. ∼150K IDR-reproducible peaks from TFAP2C, TEAD4, CDX2, YAP1, and GATA3 ChIP-nexus experiments were pooled and used as model inputs. Validation datasets were peaks across chr5,6,7,19; test datasets were peaks across chr1,8,9, and peaks across chrX and Y chromosomes were excluded from the analysis. The remaining regions were used for model training. Hyper-parameters were the default BPNet architecture. The trained model performance was assessed by comparing (1) area under the PrecisionRecall Curves (auPRC) for profiles over different bins of resolution between observed ChIP-nexus profiles and predicted BPNet profiles (Figure S1C) (2) Jensen-shannon distance for TF binding profile between observed ChIP-nexus signals to predicted BPNet signals for each TF and (3) counts correlations of observed ChIP-nexus signals to predicted BPNet signals for each TF (Figure S1E) as previously described.^15^ The auPRC values were benchmarked alongside replicate-replicate, observed random, and observed-average observed profile comparisons to establish an in-context understanding of predicted profile accuracy. All BPNet models were implemented and trained using Keras (v2.2.4), TensorFlow1 backend (v.1.70), and the Adam optimizer150. The training used an NVIDIA TITAN RTX GPU with CUDA v9.0 and cuDNN v7.0.5 drivers. To obtain the *Tead double* motifs in ESCs for analysis Figure 5G, TEAD4 ChIP-nexus experiments were pooled and used as model inputs to train a single TF model; ∼15K IDR-reproducible peaks were used. Validation peak datasets across chr 1,7,3,14, test peak datasets across chr2,8,9, and peaks across chromosomes X and Y were excluded from the analysis. Hyper-parameters, model performances, and BPNet implementation were performed as described above. PAtCh-Cap control in ESCs was from.^15^ We performed DeepLIFT and TF-MoDISco on the trained model to generate an ESCs-specific *Tead motif* set. For analysis in Figure 5G, we used *Tead double* motifs from fold 1. Additional models were trained with the same architecture as part of 3-fold validation (fold 2 and fold 3). Spearman counts correlation values (top right) were determined by comparing the observed ChIP-nexus counts with BPNet’s predicted counts at TEAD4 ChIP-nexus peaks in ESCs (Figure S5I).

#### Motif extraction, curation, and island generation

DeepLIFT (v0.6.9.0, derived from the Kundaje Lab fork of DeepExplain (https://github.com/kundajelab/DeepExplain)^63^ was applied to the trained BPNet model to generate the contribution of each base across a given input sequence to the predicted output counts and profile signals. Contribution scores for counts and profile outputs were generated for all 5 TF tasks. TF-MoDISco (v0.4.2.2)^64^ was then applied for each TF separately. Regions of high counts contribution were identified, clustered based on within-group contribution and sequence similarity, and then consolidated into motifs. The *Tfap2c, Tead, Cdx2, Gata3, and Yap1* motifs were manually identified based on their similarity to the known motif and the sharp average ChIP-nexus binding footprint of the corresponding TF. Once motifs were characterized and confirmed, they were used to label genomic instances by CWM scanning as previously described.^15^ Briefly, a motif was mapped based on both Jaccardian similarity to the TF-MoDISco contribution weight matrix (CWM) and sufficient total absolute contribution across the mapped motif. Then, motifs were filtered for redundant assignment of palindromic sequences and overlapping peaks. To obtain regions of mapped motif combinations with enhancers for downstream measurement of enhancer activity to get specific mapped motif pairs, motif islands were generated as described.^20^ Each island starts as a 500 bp (enhancer window) region centered on the motif and gets clustered and merged with another nearby motif island if they overlap. In this manner, islands get extended if a motif is within less than 500 bp. The motif islands, by their motif combinations with motif numbers, read sums of TFs binding and enhancer activity (provided in Table S4).

#### Visualization of YAP1 binding and enhancer activity markers

To visualize the correlation between YAP1 binding and the markers of enhancer activity, we selected regions using the following criteria: BPNet-mapped motifs that were absent of ERVs, were within TEAD4 peaks, and showed TEAD4 binding. At those regions, we calculated the total ChIP-nexus read counts for YAP1, selected regions above the median value, and sorted based on the total read counts. These regions were then divided into the top 5000 regions with high YAP1 reads and the 5000 regions with median YAP1 binding. We used this set to calculate normalized reads and generate the TEAD4, YAP1, H3K27ac, Pol II, and Nascent-RNA heatmap.

#### Motif pair interaction analysis

We selected mapped regions with only one motif pair from the motif islands set for the following motif-pair combinations: *Tfap2c-Tead, Cdx2-Tead, Gata3-Tead4, and Tfap2c-Tead double*. We then sorted the regions by the distance between the two motifs and included distances less than 160 bp for display. YAP1 contribution scores from the binding model were used to make heatmaps in ggplot. The *in silico* motif interaction analysis and odds ratio calculations for the co-occurrence likelihood of motif pairs were performed as described.^15^ Statistics for Figures 3C, S3C, and S3D provided in Table S5 and described in STAR Methods.

Yap1 binding enhancement was calculated by measuring BPNet model predictions across computationally generated sequences where we injected two motifs–a *Tead single* motif and another motif (y axis on 1G) into 256 randomized sequences. We next averaged predictions across these 256 trials. More precisely, this *in silico* motif interaction analysis was performed to measure “YAP1 binding enhancement” as described previously.^15^ When injecting two motif sequences (motif A and motif B) across motif pair distances (*d*) ranging up to 150 bp. Yap1 binding predictions were measured in these different simulation scenarios where the sum of the read counts predicted across a 50 bp window centered on motif A. We measured four different cases: (1) neither motif A nor motif B was injected into the sequence (*hØ*), (2) motif A only was injected into the sequence (*hA*), (3) motif B only was injected into the sequence (*hB*), and (4) motif A and motif B were both injected into the sequence at a designated distance (*hAB*). After each case was measured across all motif combinations and distances, then averaged across trials, the *in silico* binding enhancement for each motif in a motif pair was calculated using the following equation: log2((*h*AB *–* (*h*B – *hØ*))/*h*A). The motif pairs considered were combinations of the highest affinity sequence representations of *Tead single* (ACATTCCTG), *Tfap2c* (CCCTCAGGC), *Cdx2* (GCCATAAA), *Gata3* (AGATAAG), *Jun-Fos* (ATGAGTCAT), *CTCF* (CCACTAGGGGGCG), *Elf5* (CCGGAAG), *Gata3 double* (AGATAAGATCT) and *Tead double* (ACATTCCTGGCATTCC).

#### Enhancer regions selection for reporter assay

This analysis was to predict TFAP2C and TEAD4 binding on genomic regions with different motif distances and how this changes upon editing the distance between the motifs. From our islands, we selected regions with one Tead single and one *Tfap2c* motif within less than 200 bp and resized them to 400 bp putative enhancers, and recorded the coordinates of *Tfap2c* and *Tead4* motifs within the putative enhancers for mutations. We then identified the nearest genes using the biomaRt package. For each putative enhancer, we generated sequences for wild-type, mutated motif for each at its original position by mutating the two most contributing nucleotides to the least contributing within that motif. Then, we inserted the same motif at distances in multiples of 10 or 15 within a 400 bp window. These sequences were combined into an array to predict TF binding and contributions at a motif range of 50 bp. The resulting unique enhancer values were exported in R for plotting. The luciferase assay and CRISPR regions were selected by high binding of TEAD4, TFAP2C, and YAP1 at these putative enhancers and by context-relevant gene targets.

#### Extracting regions with different *Tead double* motif spacings

To map regions in the mm10 genome with different *Tead double* motif spacings, we used pattern matching (with no mismatches) to identify instances of a single Tead motif (RMATTCCWD). Then, regions with two motifs within 23 bp were identified, and the frequencies by which each motif spacing occurred were recorded. Thus, for a motif spacing of 2, the matched sequence is RMATTCCNNRMATTCCNN. The predicted TEAD4 binding signal was then calculated for all motifs injected into randomized sequences and averaged over 256 iterations. The results from each spacing were then averaged.

#### *Tead* motifs variant analysis

To assess the distribution of motif variant frequency, identical sequence patterns of CWM-mapped *Tead single* and *double* motif patterns were grouped, analyzed and visualized. To obtain a robust representation, only patterns that occurred in the top 90th percentile and occurred at least 10 times were considered. After injecting each sequence pattern into 256 random sequences, BPNet was used to predict TEAD4 binding. The average predicted signal for each pattern, along with the pattern frequency, was plotted using ggplot.

#### Genome-wide TEAD4 binding cooperativity on *Tead double* motifs

This analysis aimed to investigate the potential synergy between each side (each *Tead* motif) of the *Tead* double motifs. We selected regions that did not overlap with either ERVs or promoter regions, extracted the sequences of the *Tead double* motifs, and oriented them in the 5’>3′ direction. We then split the motif sequences into two half-sites, each corresponding to a *Tead single* motif. We then predicted the binding of TEAD4 at the half-sites and the complete double motifs injected into random sequences. The values for the two half-sites were summed and compared to those for the complete double motifs as a measure of synergy between the two half-sites of TEAD4 double motifs.

#### Benchmarking motif discovery and analysis

This analysis aimed to benchmark the motif discovery and scanning approaches leveraging PWMs (frequency-based) versus CWMs (contribution-based) for analyzing *Tead* motifs. We performed traditional PWM scanning, leveraging MEME (5.3 and 5.5.3) and FIMO (5.5.3)^137^^,^^162^ We ran MEME (*-mod anr*) on the top 1,000 TEAD4 ChIP-nexus peaks within the central 101 bp regions of each peak summit, returning both a *Tead* single and *Tead* double motif. Using FIMO (--skip-matched-sequence --parse-genomic-coord --max-strand --max-stored-scores 10000000), we performed two rounds of motif scanning. The first round was conducted on *Tead* single and *Tead* double motif PWMs returned by MEME, described above (called “PWM-freq”). The second round was conducted on *Tead* single and *Tead* double motif PWMs reconstituted from seqlets returned by TF-MoDISco (called “CWM-freq”). We compared each set of FIMO-mapped motifs, alongside the motifs mapped using CWM-scanning, described above (called “CWM-contrib”) (Figure 4C). This resulted in three groups of mapped motifs.

For the three groups of motif mappings (PWM-freq, CWM-freq, and CWM-contrib), we scored the sequence match of each motif from each mapping group using the CWM-freq PWM as a reference. We next performed in silico analysis of each mapping group, taking their respective top 5K *Tead* double motifs and top 20K *Tead* single motifs based on the sequence-match score described above. We injected each unique motif sequence from these top-scoring motifs into randomized DNA sequences and predicted TEAD4 binding. We evaluated genomic context influencing TEAD4 binding predictions by predicting genomic regions that mapped CWM-contrib motifs labeled as “CWM-contrib in genomic context.”

We performed additional benchmarking of CWM-versus PWM-scanning by leveraging an alternative motif mapping software called HOMER (4.9.1)^163^ to identify motifs. Run under default parameters of fragment size of 200 bp and a variety of allowed motif lengths (i.e., 9, 13, 18, 20 bp) from the top 1000 TEAD4 ChIP-nexus peaks, HOMER returned a matched *Tead single* motif, not the double motif. However, when we looked at the list of *de novo* motifs similar to the *Tead single* motif, we could identify the *Tead double* motif by specifically searching for the pattern (Figure S4A). Given the long list, it is unlikely that the motif would stand out to someone not looking for it.

## Supplemental information

Document S1. Figures S1–S6 and Table S1

Table S2. Table of crRNA and oligos used in CRISPR-Cas9 experiments, related to STAR Methods

Table S3. Table on luciferase assay, related to STAR Methods

Table S4. Mapped motif combinations with total reads of enhancer activity markers across islands, related to Figure 2 and STAR Methods

Table S5. Statistics on distance-dependent change in contribution scores of the heatmaps, related to Figures 3C, S3C, and S3D and STAR Methods

Document S2. Article plus supplemental information
