# Supplementary material for: Interpreting regulatory mechanisms of Hippo signaling through a deep learning sequence model
Source: Cell Genom. 2025 Apr 1;5(4):100821. doi: 10.1016/j.xgen.2025.100821 (PMC12008814; doi:10.1016/j.xgen.2025.100821)
Supplement: Document S1. Figures S1–S6 and Table S1 [file mmc1.pdf]

Cell Genomics, Volume *5*

Supplemental information

Interpreting regulatory mechanisms of Hippo signaling through a deep learning sequence model

Khyati Dalal, Charles McAnany, Melanie Weilert, Mary Cathleen McKinney, Sabrina Krueger, and Julia Zeitlinger

Supplementary Figures

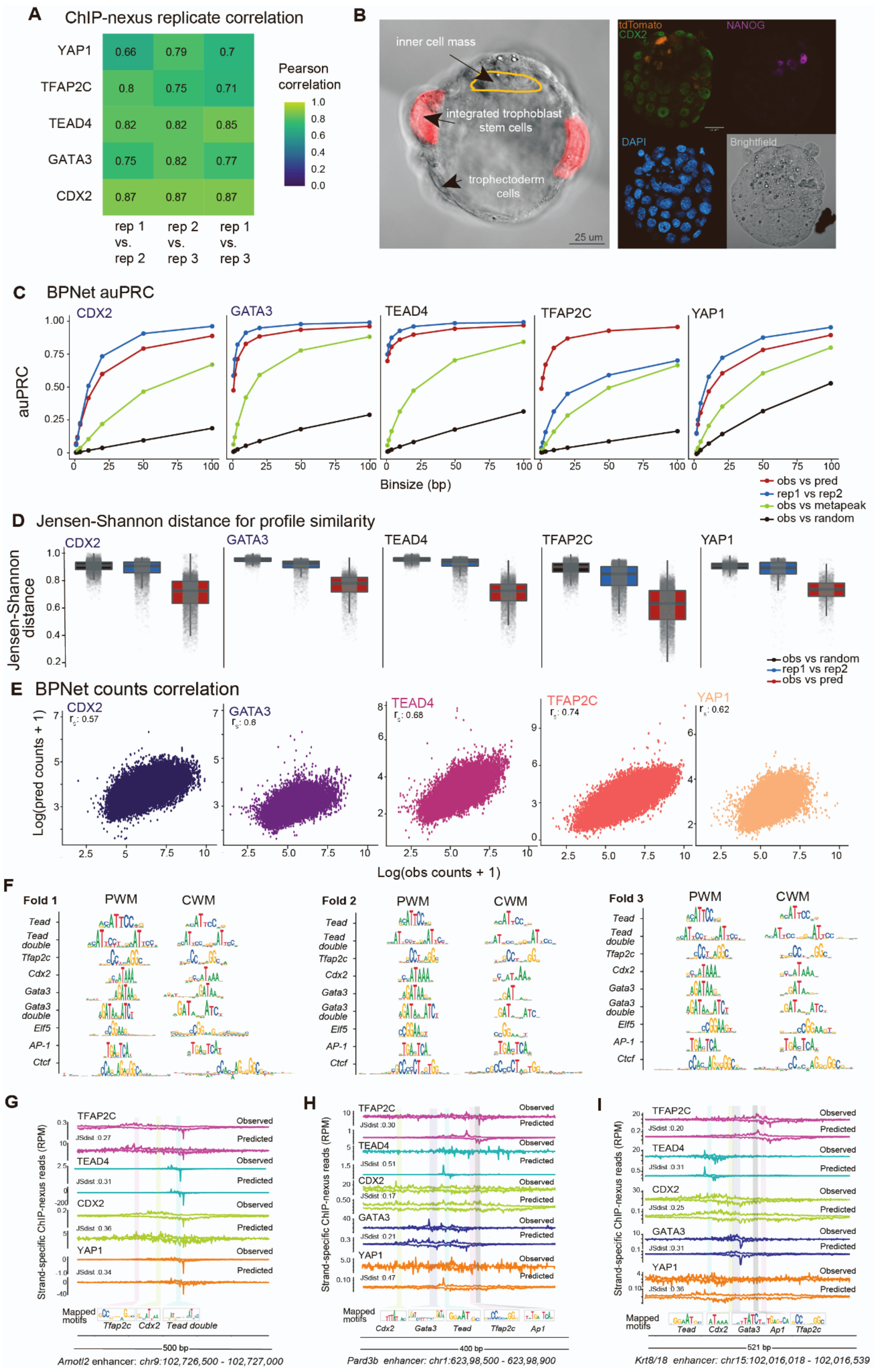

Figure S1 | BPNet accurately learns the profile and counts information for TFs important in TSCs (related to Figure 1)

A) Pairwise comparisons with Pearson correlation values of TF ChIP-nexus experiments between the three replicates. The coverage for each replicate was calculated across a 200bp window centered on the MACS2-called peaks for each TF. Because ChIP-nexus provides strand-specific information, the counts from both strands were added. **B**) Using an aggregation assay at the blastocyst stage of mouse embryos, TSCs expressing a td-Tomato lentivirus construct get integrated into the outer trophectoderm (TE) layer cells, thus closely resembling the fate of the neighboring cells from where they were derived (left). Immunofluorescence staining on an aggregated embryo reveals that td-Tomato cells preferentially express CDX2, a marker for the trophectoderm layer, rather than NANOG, a marker for the inner cell mass (right). **C**) Area under the Precision-Recall Curves (auPRC) shows that BPNet accurately predicts the ChIP-nexus profile peak positions, assessed at various resolutions up to 100 bp. Replicate experiments, average ChIP-nexus profiles, and randomized profiles are shown as controls. **D**) The similarities between observed and predicted profiles at ChIP-nexus peaks on the withheld chromosomes were quantified by Jensen-Shannon (JS) distance for each TF (0=perfect concordance, 1=no similarity). **E**) BPNet accurately predicts ChIP-nexus counts at ChIP-nexus peaks on the withheld chromosomes. Spearman counts correlation values were determined for each TF by comparing the observed ChIP-nexus counts (x axis) with BPNet's predicted counts (y axis) . **F**) Representative short motifs discovered with TF-MoDISco for three different folds, trained with the same architecture . All sequence logos share the same *y-axis*. **G-I**) Comparison of experimentally generated TF binding with BPNet-predicted TF binding at the putative (G) *Amotl2* enhancer, (H) *Pard3b* enhancer, and (I) *Krt8/18* enhancer illustrates BPNet's predictive accuracy. Each color is a different TF, where the top track is the experimental ChIP-nexus data, and the bottom track is the predicted binding. BPNet-mapped motifs are shown below. Putative *Amotl2* and *Pard3b* enhancers were on the withheld chromosome during BPNet training. Note the fuzzy profile of YAP1, an indirect binder, and the the predicted YAP1 profile that looks denoised The similarities between observed and predicted profiles at the specific region, quantified by Jensen-Shannon (JS) are displayed for each TF (the lower the value, the better the correlation).

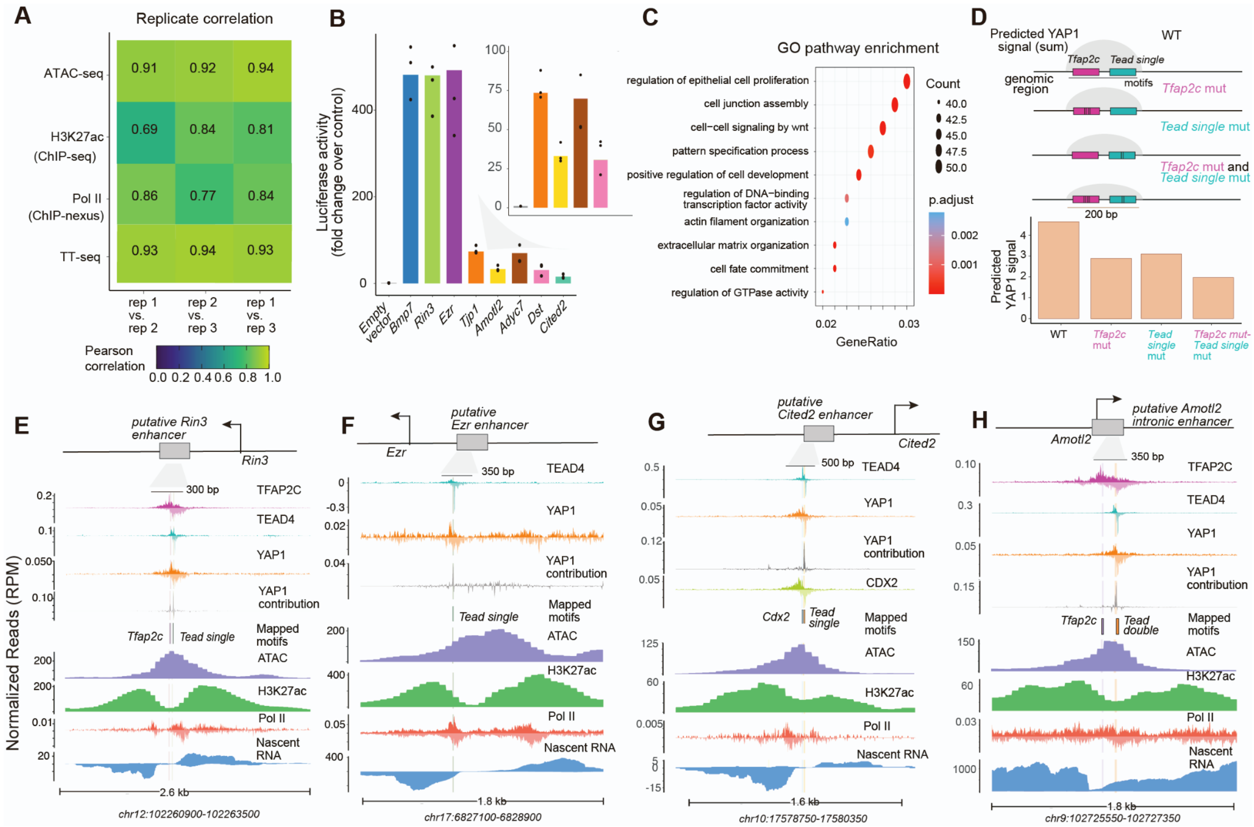

Figure S2 | TSCs specific enhancers show activity markers with TFs bound motifs (related to Figures 2 and 3)

A) Pearson correlation values were determined for all pairwise comparisons between the three replicates of ATAC-seq, H3K27ac ChIP-seq, Pol II ChIP-nexus, and TT-seq experiments. For Pol II ChIP-nexus, the coverage for each replicate was calculated across a 200bp window centered on the MACS2-called peaks. For TF ChIP-nexus experiments between the three replicates. The coverage for each replicate was calculated across a 200bp window centered on the MACS2-called peaks for each TF. Because ChIP-nexus provides strand-specific information, the counts from both strands were added. For ATAC-seq, counts for each replicate were calculated across a 600bp window centered on the MACS2-called peaks. For ChIP-seq, counts for each replicate were calculated across a 1000bp window centered on the MACS2-called peaks. For TT-seq, counts for each replicate were calculated across a 500bp window centered on the MACS2-called peaks of Pol II. **B**) Luciferase assay of the wild-type 175bp or 200bp minimal putative enhancers consisting of either the *Tead single* and *Tfap2c* motif pair or the *Tead double motif* was performed in three biological replicates and normalized over the empty vector control. **C**) The *Tead single* and *Tfap2c* motif-pair islands mapped within 160bp distance were used to find the nearest gene for performing gene ontology analysis with the clusterProfiler package. **D**) Average YAP1 predicted signal summed across a 200bp window within the putative *Bmp7* enhancer (portrayed as grey in the graphic) for the wild-type *Tead single* and *Tfap2c* motif, and when individual motifs or both motifs are mutated. A strong reduction is already seen when one motif is mutated, suggesting cooperativity between the two motifs. **E-H**) The genome track of active enhancers for the putative target genes E) *Rin3*, F) *Ezr*, G) *Cited2*, H) *Amotl2* with mapped motifs, normalized ChIP-nexus TFs binding profiles, and normalized read pileups of enhancer activity markers.

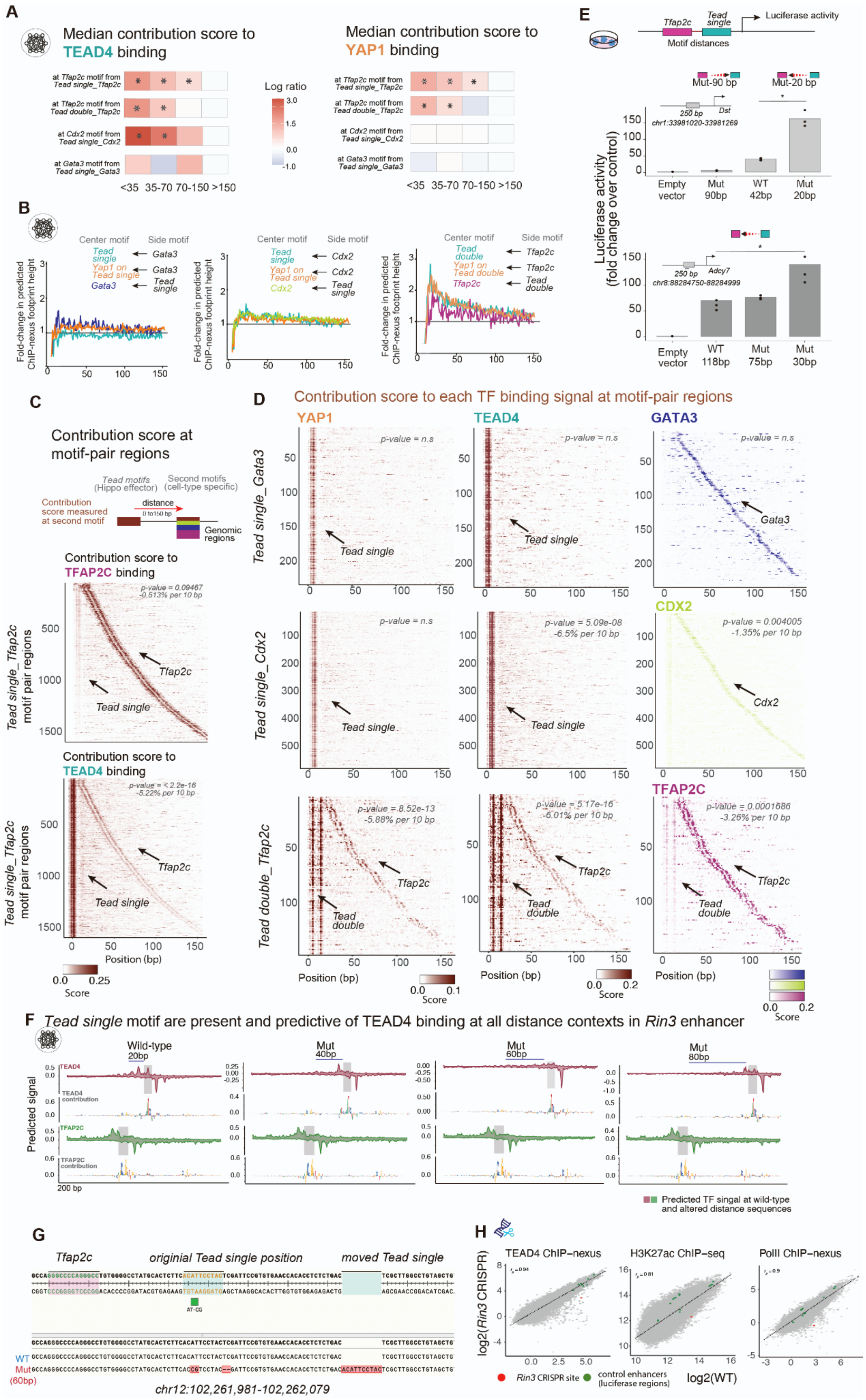
A
Median contribution score to TEAD4 binding
Median contribution score to YAP1 binding
at Tfap2c motif from Tead single_Tfap2c
at Tfap2c motif from Tead double_Tfap2c
at Cdx2 motif from Tead single_Cdx2
at Gata3 motif from Tead single_Gata3
Log ratio
<35 35-70 70-150 >150
B
Center motif
Side motif
Fold-change in predicted ChIP-nexus footprint height
C
Contribution score at motif-pair regions
Contribution score to TFAP2C binding
Contribution score to TEAD4 binding
Tead single_Tfap2c motif pair regions
Position (bp)
Score
D
Contribution score to each TF binding signal at motif-pair regions
YAP1
TEAD4
GATA3
CDX2
TFAP2C
Tead single_Gata3
Tead single_Cdx2
Tead double_Tfap2c
E
Luciferase activity
Motif distances
Luciferase activity (fold change over control)
Empty vector
F
Tead single motif are present and predictive of TEAD4 binding at all distance contexts in Rin3 enhancer
Wild-type
Predicted signal
Predicted TF singal at wild-type and altered distance sequences
G
Tfap2c
originial Tead single position
moved Tead single
chr12:102,261,981-102,262,079
H
TEAD4 ChIP–nexus
H3K27ac ChIP–seq
PolII ChIP–nexus
log2(Rin3 CRISPR)
log2(WT)
Rin3 CRISPR site
control enhancers (luciferase regions)

Figure S3 | The distance-dependent cooperativity of the *Tead* motif with *Tfap2c* is motif-specific and directional (related to Figure 3)

A) Identification of motifs that contribute significantly more toTEAD4 and YAP1 binding when they are in close distance to the *Tead* motifs, denoted by $*p < 5e{-}5$ in a Wilcoxon test when comparing to baseline scores where the motif is >150bp away. For each motif, the median log ratio of the contribution scores over the baseline is shown for four distance intervals. Note that *Cdx2* motifs appear to significantly influence TEAD4 binding in a distance-dependent manner; however, as shown below, the levels are so low that the change is likely due to background. **B**) In the *in silico* analysis, motifs are injected into randomized sequences, and BPNet is used to predict the average enhancement of TF binding to its motif (center) in the presence of a side motif [1]. The results show no distance-dependent TEAD4 and YAP1 binding enhancement in the presence of *Gata3* or *Cdx2* motifs. For *the Tead double-Tfap2c* motif pair, we observe mutual binding enhancement of TEAD4, YAP1, and TFAP2C at close distances. **C**) Analogous to Figure 3C showing contribution scores to YAP1 binding, these heatmaps show the contribution scores to TFAP2C and TEAD4 binding at genomic regions that were ordered by the distance between *Tead single and Tfap2c* (up to 160 bp). The contribution from the *Tead single* motif to TFAP2C binding is weak, while the contribution from the *Tfap2c* motif to TEAD4 binding is strong and decreases with larger distances, a relationship that, when modeled with linear regression, gives a slope of -5.22% per 10 bp and a p-value of <2.2e-16, confirming a statistically significant linear relationship. **D**) Heatmap showing binding contribution scores for the indicated TF (colored) at genomic regions ordered by the distance between motif pairs (up to 160 bp). Results are shown for a linear regression model between the distance and the contribution scores at the second motif (moving distally in individual heatmaps) (Table 5 and STAR Methods). **E**) Luciferase assay of the wild-type and mutated 200bp minimal putative enhancer of *Dst (*mm10-*chr1:33981020-33981269)* and *Adcy7* (mm10-chr8:88284750-88284999) were performed in three biological replicates and normalized over the empty vector control. Significance was determined by a student's t-test ($p < 0.05$). Increasing the distance between *Tead single* and *Tfap2c* decreases activity while decreasing the distance increases activity. **F**) *In silico* prediction analysis at the *Rin3* enhancer shows that the *Tead single* motif remains bound by TEAD4 at different distances from *the Tfap2c* motif. The *Tead single* motif sequence is the same at different distances, as seen in the sequence contribution scores. As a control, TFAP2C binding at *the Tfap2c* motif is minimally affected. The motif distance of 60 bp was chosen for experimental validation. **G**) Sanger sequencing was performed to confirm modifications at the putative *Rin3* enhancer with mapped and moved motifs, where the wild-type sequence has a distance of 20 bp between *Tfap2c* and *Tead single* motif. Through sequential CRISPR, a *Tead single* motif was first inserted away from the *Tfap2c* motif to generate mutant cells with a new distance of 60 bp. Then, the most important bases (highlighted in the green box) within the *Tead single* motif at the original position were mutated to abolish TEAD4 binding. **H**) Pairwise comparisons between WT and CRISPR clone cells show high Pearson correlations for TEAD4, Pol II ChIP-nexus, and H3K27ac ChIP-seq data. Control enhancer regions (those validated by luciferase assays) were unchanged (green dots)(Figure S2B), while the mutated *Rin3* enhancer region (CRISPR site in red dot) was reduced in all cases. Differential analysis by DESeq2, which takes replicates into account, was performed to obtain p-values (shown in Figure 3G).

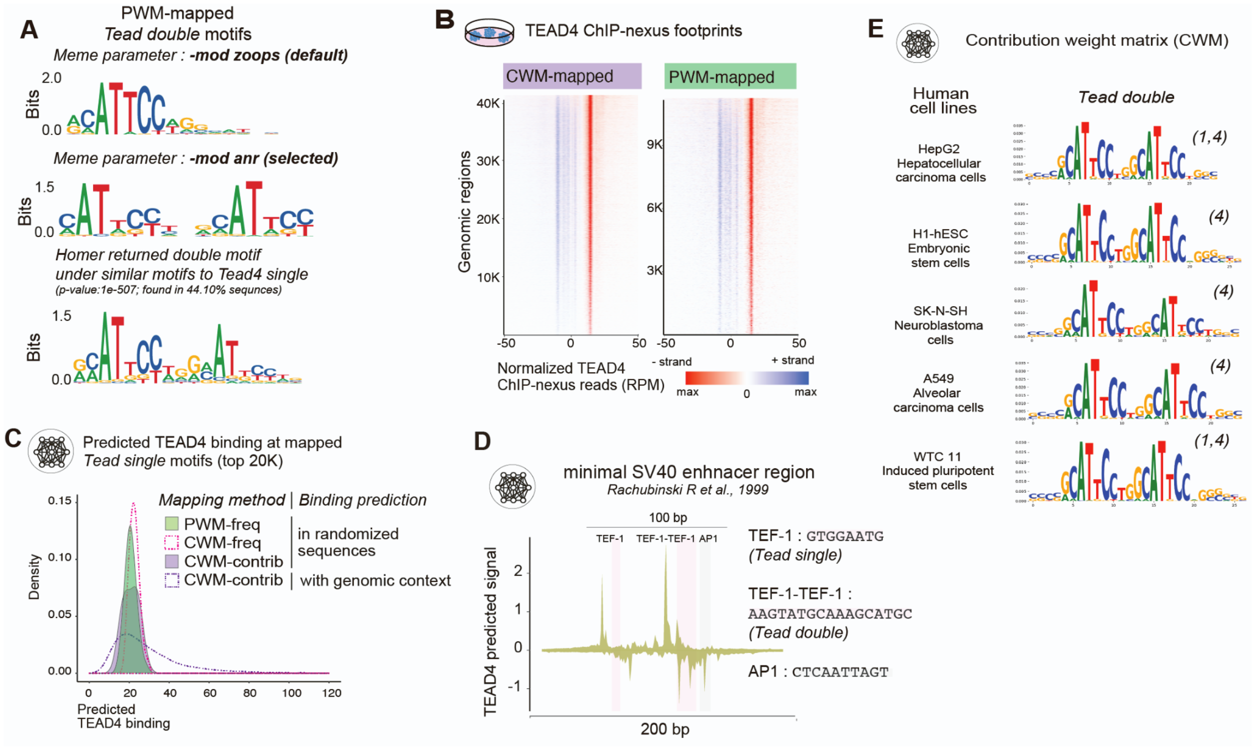

Figure S4 | BPNet approach is suited to robustly discover and map functional motifs (related to Figure 4)

A) PWM motif logos of *Tead double* motifs identified by MEME using the top 1,000 TEAD4 peaks as input and two different parameter settings. With default settings (*-mod zoops*), a motif that weakly resembles a *Tead double* motif was identified with prior knowledge of what the *Tead double* motif looks like. MEME's other setting (*-mod anr*) allows more than one non-overlapping motif to match within a single sequence, leading to the clear discovery of the *Tead double* motif. Motif discovery using HOMER primarily returned the *Tead single* motif, but the *Tead double* motif was among the similar motifs when explicitly searching for the pattern. **B**) *Tead single* motifs mapped by CWM-scanning and PWM-scanning using FIMO both show strong TEAD4 *in vivo* ChIP-nexus binding footprints; + strand (blue) and - strand (red). Regions were centered on the left side of the motifs and sorted by total ChIP–nexus binding signal. **C**) Predicted TEAD4 signal for the top 20K scoring *Tead single* motifs from each category. The frequency-based PWM-mapped motifs, the frequency-based CWM-mapped motifs (with and without using contribution scores as filter) in randomized sequences show similar predicted binding strength. In comparison, the same CWM-mapped motifs (using contribution scores) predicted in their native genomic region show a wider distribution, showing that binding to the *Tead single* motifs is strongly influenced by the surrounding sequence context. **D)** BPNet predicted TEAD4 ChIP-nexus profile at the minimal SV40 enhancer (100 bp), which contains a TEF-1 (*Tead single*) and a TEF-1-TEF-1 (*Tead double*) motif. **E)** The contribution weight matrix (CWM) of *Tead double* motifs of either human TEAD4, TEAD1, or both (shown in brackets) obtained from training TF ChIP-seq data in various human cell lines from the ENCODE Consortium. The data were generated by the lab of Richard Myers, HAIB, with the following identifiers: ENCSR934WOF; ENCSR497JLX, ENCSR285HHZ, ENCSR800JRG, ENCSR000BUQ, ENCSR000BRY, ENCSR000BUD. Single BPNet models for each experiment were trained by Anshul Kundaje's lab at Stanford.

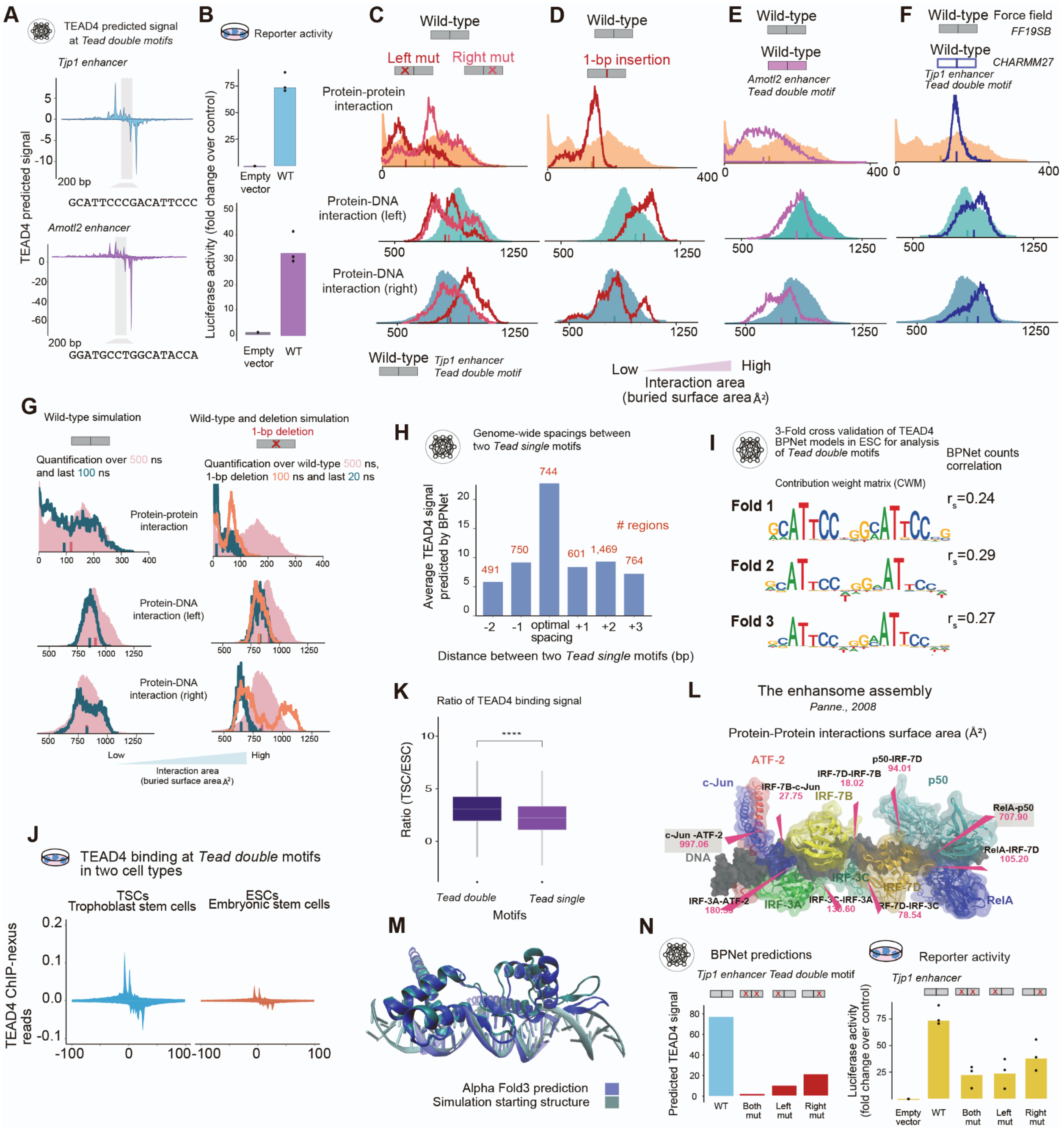

Figure S5 | MD simulations reveal the labile nature of TEAD4 cooperative binding at *Tead double* motifs (related to Figure 5)

A) BPNet predicts strong TEAD4 footprints on the putative enhancers of *Tjp1* and *Amotl2* harboring a *Tead double* motif. **B)** Luciferase assay results for the 200bp minimal *Tjp1* enhancer (mm10-chr7:65,430,487-65,430,686) and the 175bp minimal *Amotl2* enhancer (mm10-chr9:102,726,395-102,726,570), normalized over the empty vector control, performed in three biological replicates. **C**) Buried surface area distributions for simulations using the wild-type *Tead double* motif from the *Tjp1* enhancer (solid area) and two mutations (red lines). The protein-protein interactions display an asymmetric response to the two mutations, and this response was validated (Figure S5N). **D)** Buried surface area distribution for the 1-bp insertion show a narrower distribution that never reaches the high (>200 Å^2) interaction areas of the high-affinity case. **E**) An MD simulation with the *Tead double* motif from the putative *Amotl2* enhancer (highlighted in pink) shows similar results except that the motif seems slightly weaker. The

protein-nucleobase interactions are slightly shifted to the left, and the protein-protein interaction becomes marginally weaker, suggesting that the whole complex is less stable than the *Tjp1 Tead double* motif (solid area). **F)** A simulation of the *Tjp1 Tead double* motif using the CHARMM27 force field gives similar results to the high-affinity simulation using FF19SB**.** CHARMM27 is known to show more limited motion than the Amber family of force fields[2] and we see a similarly narrower distribution of interaction values in our simulation. **G)** On the left side of the plot, we compared the whole simulation (500 ns) and the last 100 ns of wild-type simulation and observed good agreement between the last 100 ns (teal line) and the whole-simulation average (pink fill). On the right, we compared the full 100 ns and the last 20 ns of the 1-bp deletion simulation, and we see that the final frames show the right-side protein more detached than the early simulation. Here, the pink-filled region shows the wild-type 500 ns distribution, the orange trace shows the deletion over the whole 100 ns simulation, and the teal trace shows the distribution during the final 20 ns. These results are consistent with the protein-protein interactions being quite labile and transient and reinforce the fact that our simulations do not capture the whole equilibrium ensemble. **H)** BPNet predicts higher TEAD4 binding when two high-affinity *Tead single* motifs have the optimal spacing compared to other spacer lengths, although the optimal spacing is not more frequent (number # of regions shown in red). Predictions were performed after injecting the different motif spaced sequences into random sequences and are shown as a summed signal in a 50bp window of the injected motif averaged across all random sequences. **I)** The *Tead double* motifs were discovered with TF-MoDISco for ESCs with additional models trained with the same architecture as part of a three-fold validation (fold 2 and fold 3). Spearman counts correlation values (top right) were determined by comparing the observed ChIP-nexus counts with BPNet's predicted counts at TEAD4 ChIP-nexus peaks in ESCs. **J)** The average observed (and predicted) TEAD4 binding footprint at the mapped *Tead double* motifs in two cell types. *Tead double* motifs (TSCs:- ~14k and ESCs:- ~1k) were from fold 1 of their respective trained models. **K)** The relative binding ratio of TEAD4 in trophoblasts relative to embryonic stem cells is significantly higher for *Tead double* motifs than for *Tead single* motifs (Wilcoxon test, $p < 0.0001$). This is consistent with a stronger stabilization of TEAD4 on the *Tead double* motif in the presence of YAP1 and TAZ. **L)** The predicted enhanceosome structure[3] shows mostly weak interactions that are likely transient, with only two pairs of TFs (highlighted in the grey box) having a buried surface area over 200 $Å^2$. These weak interactions are of a similar magnitude to the 120 $Å^2$ buried surface area between the two Tead4 proteins in the wild-type simulations (B, C, D, orange fill). **M)** Comparison of the structure we used as the starting point for our simulations with an AlphaFold3-based model of two TEAD4 molecules bound to a *Tjp1* high-affinity *Tead double* motif. A STAMP alignment[4] between two structures shows that they are highly similar. **N)** BPNet predicted TEAD4 binding on the *Tjp1 Tead double motif* (GCATTCCCGACATTCCC) and on the same sequence where either the left (GCGGTCCCGACATTCCC), the right (GCATTCCCGACCGTCCC), or both (GCGGTCCCGACCGTCCC) *Tead single* motif components are mutated. Predictions were performed after injecting the motif sequences into random sequences, and the summed signals in a 100 bp window of the injected motif were averaged across all random sequences. On the right, a normalized luciferase assay consisting of the motif or its mutant variants was performed as three biological replicates, as highlighted in Figure 5B, and used for MD simulations in Figure S5C**.**

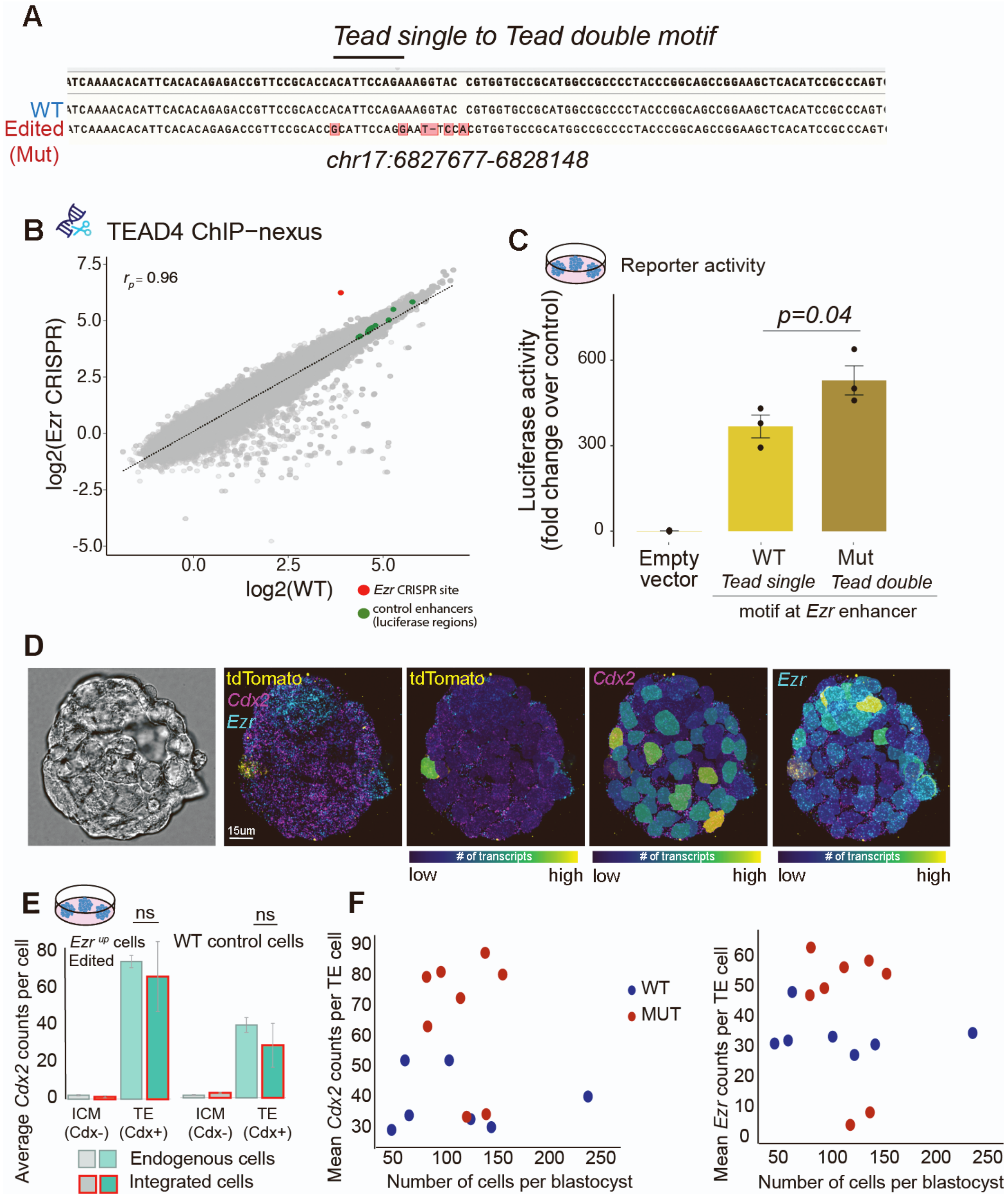

Figure S6 | The edited *Tead double* motif within *the Ezr* enhancer shows increased TEAD4 binding, activity, and cell-specific gene expression (related to Figure 6)

A) Sanger sequencing confirmed the modification at the *Ezr* enhancer, where the wild-type (WT) *Tead single* motif was edited into the *Tead double* motif via homology-directed CRISPR-Cas9 (Mut). **B**) Pairwise comparisons between WT and CRISPR clone cells show high Pearson correlations for the TEAD4 ChIP-nexus data. Control enhancers (those validated by luciferase assay in Figure S2B) shown as green dots remain unaltered, while the mutated *Ezr* enhancer region (CRISPR site) shown as red dot is strongly increased. Differential analysis using DESeq2 was performed to obtain significance values (red dot: p-value= 2.34e-07, not significant for the green dots). **C**) Luciferase assay of the wild-type and mutated 200bp minimal *Ezr* enhancer were performed in three biological replicates and normalized to the empty vector control. Significance was determined by a student's t-test ($p<0.05$). **D**) HCR-FISH was performed on aggregated mouse blastocyst embryos with wild-type (WT) or mutant (edited Ezr^{up}) cells for probes *Cdx2, Ezr,* and

td-Tomato for quantification. The nuclei masks were made with Cellpose and Napari software using the DAPI channel, which was used on other channels to quantify average *Cdx2* counts to distinguish cells between inner cell mass and trophectoderm layer (shown in E), and td-Tomato stain was used to distinguish between native vs aggregated cells. **E**) Average quantification of *Cdx2* counts. Student's t-test was performed between endogenous and integrated cells of edited Ezr^{up} and wild-type cell population (p > 0.05).Error bars show standard error of the mean (SEM). **F**) The aggregated embryos with wild-type cells show an overall lower expression of average *Cdx2 or Ezr* expression with respect to embryo size (number of cells per blastocyst) than mutant (edited Ezr^{up}) cells. All quantification was made per blastocyst to account for differences in expression.

Table S1 | Oligonucleotides used for ChIP-nexus (related to STAR Methods).

Name	Identity	Modification	Barcode	Sequence
Nex_adapter_U	Adaptor: universal	5' phosphate	/	/5Phos/GATCGGAAGAGCACACGTCTGGATCCACGACGCTCTTCC
Nex_adapter_1	Adaptor: barcoded	5' phosphate	AGTCNNNNN	/5Phos/AGTCNNNNNAGATCGGAAGAGCGTCGTGGATCCAGACGTGTGCTCTTCCGATCT
Nex_adapter_2	Adaptor: barcoded	5' phosphate	CAGTNNNNN	/5Phos/CAGTNNNNNAGATCGGAAGAGCGTCGTGGATCCAGACGTGTGCTCTTCCGATCT
Nex_adapter_3	Adaptor: barcoded	5' phosphate	GTCANNNNN	/5Phos/GTCANNNNNAGATCGGAAGAGCGTCGTGGATCCAGACGTGTGCTCTTCCGATCT
Nex_adapter_4	Adaptor: barcoded	5' phosphate	TCAGNNNNN	/5Phos/TCAGNNNNNAGATCGGAAGAGCGTCGTGGATCCAGACGTGTGCTCTTCCGATCT
Nex_primer_U	Primer: universal	3' phosphoro-thioate bond	/	AATGATACGGCGACCACCGAGATCTACACTCTTTCCCTACACGACGCTCTTCCGATC*T
Nex_primer_01	Primer: indexed	3' phosphoro-thioate bond	ATCACG	CAAGCAGAAGACGGCATACGAGATCGTGATGTGACTGGAGTTCAGACGTGTGCTCTTCCGATC*T
Nex_primer_02	Primer: indexed	3' phosphoro-thioate bond	CGATGT	CAAGCAGAAGACGGCATACGAGATACATCGGTGACTGGAGTTCAGACGTGTGCTCTTCCGATC*T
Nex_primer_03	Primer: indexed	3' phosphoro-thioate bond	TTAGGC	CAAGCAGAAGACGGCATACGAGATGCCTAAGTGACTGGAGTTCAGACGTGTGCTCTTCCGATC*T
Nex_primer_04	Primer: indexed	3' phosphoro-thioate bond	TGACCA	CAAGCAGAAGACGGCATACGAGATTGGTCAGTGACTGGAGTTCAGACGTGTGCTCTTCCGATC*T
Nex_primer_05	Primer: indexed	3' phosphoro-thioate bond	ACAGTG	CAAGCAGAAGACGGCATACGAGATCACTGTGTGACTGGAGTTCAGACGTGTGCTCTTCCGATC*T
Nex_primer_06	Primer: indexed	3' phosphoro-thioate bond	GCCAAT	CAAGCAGAAGACGGCATACGAGATATTGGCGTGACTGGAGTTCAGACGTGTGCTCTTCCGATC*T
Nex_primer_07	Primer: indexed	3' phosphoro-thioate bond	CAGATC	CAAGCAGAAGACGGCATACGAGATGATCTGGTGACTGGAGTTCAGACGTGTGCTCTTCCGATC*T
Nex_primer_08	Primer: indexed	3' phosphoro-thioate bond	ACTTGA	CAAGCAGAAGACGGCATACGAGATTCAAGTGTGACTGGAGTTCAGACGTGTGCTCTTCCGATC*T

ChIP-nexus adapters are generated using the universal adapter (Nex_adapter_U) and the barcoded adapters (Nex_adapter_1, Nex_adapter_2, Nex_adapter_3, Nex_adapter_4). The universal (Nex_primer_U) and indexed (Nex_primer_01 - Nex_primer_08) primers are used for amplification of the library. Oligonucleotides can be ordered from Integrated DNA Technologies (IDT).

Supplemental References

1. Avsec, Ž., Weilert, M., Shrikumar, A., Krueger, S., Alexandari, A., Dalal, K., Fropf, R., McAnany, C., Gagneur, J., Kundaje, A., et al. (2021). Base-resolution models of transcription-factor binding reveal soft motif syntax. Nat. Genet. *53*, 354–366. 10.1038/s41588-021-00782-6.

2. Minhas, V., Sun, T., Mirzoev, A., Korolev, N., Lyubartsev, A.P., and Nordenskiöld, L. (2020). Modeling DNA Flexibility: Comparison of Force Fields from Atomistic to Multiscale Levels. J. Phys. Chem. B *124*, 38–49. 10.1021/acs.jpcb.9b09106.

3. Panne, D. (2008). The enhanceosome. Curr. Opin. Struct. Biol. *18*, 236–242. 10.1016/j.sbi.2007.12.002.

4. Russell, R.B., and Barton, G.J. (1992). Multiple protein sequence alignment from tertiary structure comparison: assignment of global and residue confidence levels. Proteins *14*, 309–323. 10.1002/prot.340140216.
